# Perspectives in Immunotherapy: meeting report from Immunotherapy Bridge (Naples, November 30th–December 1st, 2022)

**DOI:** 10.1186/s12967-023-04329-7

**Published:** 2023-07-20

**Authors:** Paolo A. Ascierto, Antonio Avallone, Carlo Bifulco, Sergio Bracarda, Joshua D. Brody, Leisha A. Emens, Robert L. Ferris, Silvia C. Formenti, Omid Hamid, Douglas B. Johnson, Tomas Kirchhoff, Christopher A. Klebanoff, Gregory B. Lesinski, Anne Monette, Bart Neyns, Kunle Odunsi, Chrystal M. Paulos, Daniel J. Powell, Katayoun Rezvani, Brahm H. Segal, Nathan Singh, Ryan J. Sullivan, Bernard A. Fox, Igor Puzanov

**Affiliations:** 1 Department of Melanoma, Cancer Immunotherapy and Innovative Therapy, Istituto Nazionale Tumor IRCCS “Fondazione G. Pascale”, Naples, Italy; 2 Experimental Clinical Abdominal Oncology Unit, Istituto Nazionale Tumori-IRCCS-Fondazione G. Pascale, Naples, Italy; 3 Translational Molecular Pathology and Molecular Genomics, Earle A. Chiles Research Institute, Providence Cancer Institute, Portland, OR USA; 4 Department of Oncology, Medical and Translational Oncology, Azienda Ospedaliera Santa Maria, Terni, Italy; 5 Tisch Cancer Institute, Marc and Jennifer Lipschultz Precision Immunology Institute, Icahn School of Medicine at Mount Sinai, New York, NY USA; 6 UPMC Hillman Cancer Center, University of Pittsburgh, Pittsburgh, PA USA; 7 Ankyra Therapeutics, Cambridge, MA USA; 8 Weill Cornell Medicine, New York, NY USA; 9 The Angeles Clinic and Research Institute, A Cedars-Sinai Affiliate, Los Angeles, CA USA; 10 Department of Medicine, Vanderbilt University Medical Center, Nashville, TN USA; 11 Laura and Isaac Perlmutter Cancer Center, New York University (NYU) School of Medicine, NYU Langone Health, New York, NY USA; 12 Memorial Sloan Kettering Cancer Center, New York, NY USA; 13 Parker Institute for Cancer Immunotherapy, San Francisco, CA USA; 14 Department of Hematology and Medical Oncology, Emory University School of Medicine, Atlanta, GA USA; 15 Lady Davis Institute for Medical Research, Jewish General Hospital, Montreal, QC Canada; 16 Department of Medical Oncology, University Hospital Brussel, Brussels, Belgium; 17 University of Chicago Medicine Comprehensive Cancer Center, Chicago, IL USA; 18 Department of Surgery and Department of Microbiology and Immunology, Emory University School of Medicine, Atlanta, GA USA; 19 Translational Research for Cutaneous Malignancies, Winship Cancer Institute of Emory University, Atlanta, GA USA; 20 Center for Cellular Immunotherapies, Department of Pathology and Laboratory Medicine, Abramson Cancer Center, Perelman School of Medicine, University of Pennsylvania, Philadelphia, PA USA; 21 Department of Stem Cell Transplantation and Cellular Therapy, The University of Texas MD Anderson Cancer Center, Houston, TX USA; 22 Department of Internal Medicine and Department of Immunology, Roswell Park Comprehensive Cancer Center, Buffalo, NY USA; 23 Division of Oncology, Washington University School of Medicine, Saint Louis, MO USA; 24 Melanoma Program, Massachusetts General Hospital Cancer Center, Boston, MA USA; 25 Robert W. Franz Cancer Research Center, Earle A. Chiles Research Institute, Providence Cancer Institute, Portland, OR USA; 26 Department of Medicine, Roswell Park Comprehensive Cancer Center, Buffalo, NY USA

**Keywords:** Immunotherapy, Checkpoint inhibitors, Combination therapy, Biomarkers, Tumor microenvironment, Vaccine

## Abstract

The discovery and development of novel treatments that harness the patient’s immune system and prevent immune escape has dramatically improved outcomes for patients across cancer types. However, not all patients respond to immunotherapy, acquired resistance remains a challenge, and responses are poor in certain tumors which are considered to be immunologically cold. This has led to the need for new immunotherapy-based approaches, including adoptive cell transfer (ACT), therapeutic vaccines, and novel immune checkpoint inhibitors. These new approaches are focused on patients with an inadequate response to current treatments, with emerging evidence of improved responses in various cancers with new immunotherapy agents, often in combinations with existing agents. The use of cell therapies, drivers of immune response, and trends in immunotherapy were the focus of the Immunotherapy Bridge (November 30th–December 1st, 2022), organized by the Fondazione Melanoma Onlus, Naples, Italy, in collaboration with the Society for Immunotherapy of Cancer.

## Introduction

The past decade has seen the discovery and development of novel treatments that harness the patient’s immune system and prevent immune escape due to increased understanding of the tumor immune environment. The clinical success of immune checkpoint inhibitors has dramatically changed outcomes for patients across cancer types. However, not all patients respond to immunotherapy, acquired resistance remains a challenge, and responses are poor in certain immunologically cold tumor types. This has resulted in the development of additional immunotherapy-based approaches, including adoptive cell transfer (ACT), therapeutic vaccines, and inhibitors of other immune checkpoints. Numerous clinical trials and real-world experience provide evidence of improved response and survival outcomes with multiple immunotherapy agents and different combinations in various cancers.

The use of cell therapies, drivers of immune response, and trends in immunotherapy were the focus of the Immunotherapy Bridge (November 30th–December 1st, 2022).

## Cell therapies

### TCR-T, TILs and CAR-T

There are number of potential advantages to ACT therapy compared with other immunotherapies. One is the ability to administer large numbers of highly selected cells with high avidity for tumor antigens that can recognize shared and patient-specific mutated (neo) antigens. These cells can be activated ex vivo to exhibit a potent antitumor effector function. The study and/or manipulation of administered cells can potentially result in identification of the exact cell subpopulations and effector functions required for cancer regression in vivo. In addition, ACT provides an opportunity to manipulate the host before cell transfer to provide a more favorable environment for the transferred cells.

Tumor regression after administration of endogenous tumor-infiltrating lymphocytes (TILs) can be achieved, such as the durable complete responses observed in heavily pretreated patients with metastatic melanoma [1]. Clinical activity of TIL therapy has also been reported in other cancer types, including human papillomavirus-associated cancers, cholangiocarcinoma, non-small-cell lung cancer (NSCLC), and triple-negative breast cancer (TNBC). Successful TIL therapy starts with the identification, enrichment, stimulation, and expansion of potent tumor-reactive T cells. After their infusion, persistence of the tumor antigen-specific T cells is correlated with objective clinical response [2]. Transferred T cells that persist in the blood and form memory have a phenotype consistent with a less differentiated state (CD27/CD28/IL-7 receptor-α) [3]. Less differentiated features (stem-like CD39-negative phenotype, CD27/CD28 expression and longer telomere length) by transferred TILs are also associated with persistence and response [4]. In clinical practice, a key challenge for generating TIL products is the accessibility, quantity, and quality of tumor tissue in the era of neo-adjuvant therapy. Other hurdles include the need for more efficient methods of reactive TIL enrichment/selection, especially for weakly immunogenic cancers, and closed chamber methods of expansion without exhaustion. There is also a need for rational combinations beyond interleukin (IL)-2 and programmed death (PD)-1/ cytotoxic T-lymphocyte-associated antigen (CTLA)-4 immune checkpoint blockade and to combat exhaustion and restore costimulatory functions, e.g., with gene-engineered or pharmacologic agent-treated TILs. Cost and patient accessibility to therapy will need to be addressed, as will the need for training of staff (e.g., in sample transport, practical aspects of cell administration, management of toxicities).

T cell receptor (TCR) gene transfer provides some advantages relative to TIL therapy. It allows more rapid, ‘off-the-shelf’ generation of antigen-specific T cells. Most patients have T cells of suitable quantity and quality in peripheral blood which are easily accessible, so the procedure is less invasive without the need for surgery. It can involve the introduction of novel antigen specificities not found naturally in patients and there is the potential to engineer cells to be more effective than natural cytotoxic T lymphocytes or TILs. Also, with known antigen specificity, vaccination after infusion is possible. However, many targets of TCR gene therapy have been tumor-associated self-antigens expressed at low levels by healthy tissues. For example, TCR-transduced T cells targeting carcinoembryonic antigen can mediate the regression of colorectal cancer but also induce transient but severe colitis [5]. To limit normal tissue toxicities, investigators have targeted the cancer-testis class of tumor-associated antigens (e.g., MAGE, NY-ESO-1). Targeting mutated proteins—neoantigens—may also limit off-tumor toxicity and is being widely investigated. Despite this, TCRs may possess cross-reactivity for other self-proteins which is sometimes difficult to predict. Other challenges include identifying what is “good” starting material, determining the impact of prior treatments on T cell fitness, the need for strategies to promote enhanced depth and durability of response, and tumor homing/penetration challenges. Increasing patient access to treatment is again an issue, with the need for point of care manufacturing and for cell processing to be perceived as a professional service to patients rather than a biotech product. Finally, TCR T cell recognition of intracellular proteins in cancer cells is contingent upon the processing and presentation of tumor antigen epitopes. Loss of human leukocyte antigen (HLA), antigen or antigen processing machinery will limit efficacy.

Administration of T cells genetically engineered to express chimeric antigen receptors (CARs) now represent a paradigm shift for the treatment of acute lymphoid leukemia, refractory chronic lymphocytic leukemia, select forms of B-cell non-Hodgkin lymphoma (NHL), and myeloma. Still, challenges of conventional CAR T therapy exist, including the inability to control activity post-infusion, with risk of cytokine-release syndrome (CRS), neurotoxicity, long-term B cell aplasia for CD19 CAR T, and lethal on-target, off-tumor toxicity. In addition, reliance on a single antigen target involves the risk of relapse due to antigen loss or baseline heterogeneous antigen expression. The necessity of a unique CAR T cell product for each target antigen also has high associated costs. Challenges for the field include the need to identify new clean antigens, and to effectively target common epithelial carcinomas, which account for 80–90% of cancers. Multi-antigen targeting approaches are required to reduce or eliminate antigen escape/loss and there is a need for high throughput screening/optimizing of CAR constructs. Targeting low abundance antigens (e.g., using HiT receptors) and standardization of CAR T product composition (CD4/8; memory) are also required, as are strategies promoting enhanced depth and durability of response. Tumor homing/penetration and tumor microenvironment (TME) challenges remain, and strategies to limit CAR-related toxicities are also needed. In addition, increasing patient access and managing financial toxicity remain issues. Nevertheless, the development of new technologies, reporting of encouraging clinical trial results, and a high level of enthusiasm for the CAR T cell approach by the field continues to fuel investigations and offer hope for patients with hard-to-treat cancers.

### Engineered NK cells for cancer immunotherapy

Major advantages of natural killer (NK) cells over T cells for CAR therapy include that they are allogeneic with no risk of graft-versus-host disease (GvHD) and can be manufactured ‘off-the-shelf’ with lower associated costs. Antigen recognition is mediated via innate receptors expressed on NK cells in addition to via the CAR itself, meaning that relapse through target antigen loss may be less critical after CAR NK than CAR T cell therapy. In addition, toxicity associated with CAR T therapy, i.e., CRS and neurotoxicity, have not been observed with CAR NK cell therapy.

In a first-in-human clinical trial, the safety and activity of cord blood-derived CD-19/IL-15 engineered NK cells were shown in patients with lymphoid malignancies [6]. Eight of 11 patients who were treated had a response, seven of which were a complete response. Responses were rapid and seen within 30 days after infusion at all dose levels. Infused CAR-NK cells expanded and persisted at low levels for at least one year.

Despite high response rates with CAR T cell therapy, relapses are frequent. CAR T-cells uptake surface tumor antigens via trogocytosis, an active process in which the target antigen is transferred to the surface of CAR T cells, thereby decreasing tumor antigen density and impairing the ability of CAR T cells to engage with their target [7]. This induces self-recognition and fratricide, thereby causing the depletion of CAR T cells. CAR NK cell trogocytosis also drives relapse by downregulating target antigen on tumor cells and driving NK exhaustion and fratricide [8]. However, this could be prevented by a dual-CAR system incorporating both an activating CAR against the cognate tumor antigen and an NK self-recognizing inhibitory CAR that transfers a 'don't kill me' signal to NK cells upon engagement with their TROG-positive siblings.

An important question is whether CAR NK cells can be applied beyond CD19-positive malignancies. Pre-complexing NK cells with bispecific antibodies prior to infusion may facilitate CAR-like responses by NK cells and pre-activation of NK cells with cytokines to induce a memory phenotype may enhance their persistence. IL-12/15/18 pre-activated and ex vivo-expanded cord blood-NK cells have upregulated genes related to JAK-STAT signaling and interferon (IFN)-γ response [9]. The combination of AFM13, a tetravalent bispecific antibody, and IL12/15/18 pre-activation of blood and cord blood-derived NK cells exhibited enhanced killing against CD30-epressing tumor cells. In a clinical trial of AFM13-complexed CAR-like memory cord blood-NK cells in 22 patients with refractory/relapsed CD30-positive malignancies, there were no cases of CRS, neurotoxicity or GvHD, and 17/19 metabolic responses were observed (objective response rate [ORR] 89.5%) [10].

CD70 is a promising pan-cancer antigen that is generally absent in non-lymphoid normal tissue and constitutively expressed on many hematological malignancies and a considerable number of solid carcinomas. A phase I/II clinical trial evaluating the safety and efficacy of CD70 CAR NK cells for cancer immunotherapy is underway (NCT05092451).

Treatment of glioblastoma stem cell-engrafted mice with allogeneic NK cells in combination with inhibitors of integrin or transforming growth factor (TGF)-β signaling or with TGFBR2 gene-edited allogeneic NK cells prevented NK cell dysfunction and tumor growth [11]. A phase I clinical trial with a window-of-opportunity component of engineered NK cells containing deleted TGFBR2 and NR3C1 in recurrent glioblastoma is planned (NCT04991870).

**Fig. 1:**
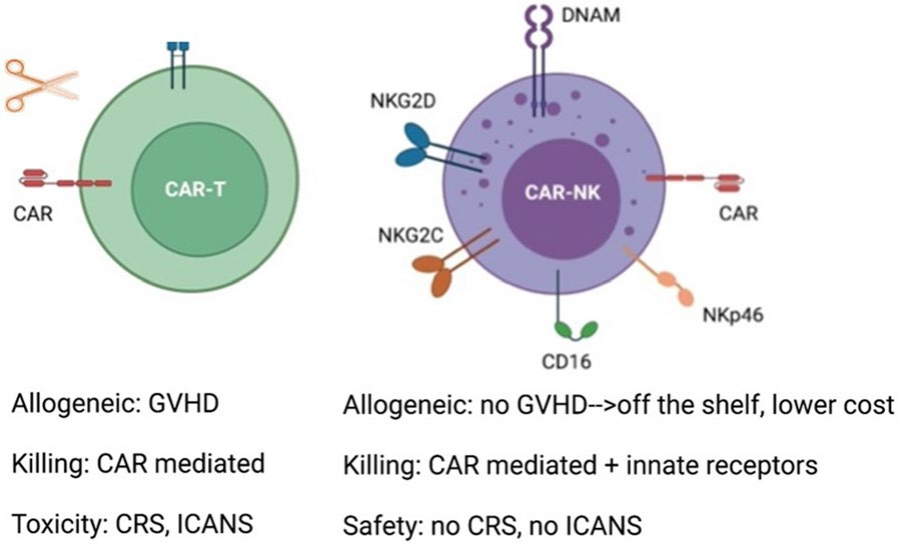
Advantages of NK cells for CAR therapy

Longer-term strategies with engineered CAR NK therapy include strategies that target more than one antigen, pre-complexing with bispecific engagers or Fc-engineered antibodies (e.g., Obinotuzumab), cytokine engineering and/or pre-activation, CRISPR gene editing, and in combination with checkpoint inhibitors, immunomodulatory drugs, or radiotherapy (Fig. 1).

### Biological drivers of CAR T cell failure

The benefits of CAR T therapy are limited in a significant number of patients with B-cell malignancies due to a lack of response after cell infusion (primary, or intrinsic, resistance) and disease relapse after initial remission (acquired resistance). Mechanisms responsible for intrinsic resistance remain poorly understood. However, inherent dysregulation of death receptor signaling leads to CAR T cell failure by impairing T cell cytotoxicity and promoting progressive CAR T cell dysfunction [12]. Pretreatment presence of complex structural variants of the protein target, APOBEC mutational signatures, and genomic damage from reactive oxygen species also predict CAR-19 resistance [13]. Acquired resistance is typically due to antigen loss, which can occur through a variety of mechanisms that span genomic mutation [14], alternative transcript splicing [15] and alteration of post-translational modifications [16].

Resistance may also be CAR T cell-driven, with intrinsic pre-infusion failure and acquired post-infusion failure. Intrinsic resistance is associated with a more differentiated T cell memory phenotype [17], and with chronic IFN signaling regulated by interferon regulatory factor 7 associated that may result in poor CAR T cell persistence [18]. The Tcf7 regulon is associated with a favorable naïve T cell state and is maintained in effector T cells in patients with long-term CAR T cell persistence. Recent correlative studies have shown that elevations in CAR T regulatory cells in either infusion products or within a few days of delivery is also associated with limited responses, suppressed CAR T cell expansion and clinical failure [19, 20]. Several recent studies have demonstrated that the biological mechanisms of CAR T cell failure may differ depending on the co-stimulatory domain contained within the CAR. While CD28-based CARs demonstrate evidence of classical T cell exhaustion, 41BB-based CARs upregulate programs directed by the transcription factor FOXO3 [21] and demonstrate phenotypic changes that resemble NK cells [22].

### Meta-analyses of immunogenomic features of response to immune checkpoint inhibition

For the eventual mainstay of immune checkpoint inhibitors (ICI) as first line therapy for benefiting cancers, the critically required robust biomarkers that may ultimately unquestionably predict responses are being extensively studied and validated in over 5600 PD-1/PD-ligand (L)-1-targeting trials worldwide [23]. An overwhelming 31,000 reports on cancer immunotherapy and biomarkers have already been published, with meta-analyses describing that more than 700 reports provide over 50 unique promising immuno-oncology biomarkers or gene signatures predicting response or resistance to experimental and FDA-approved ICI therapies [24]. Despite these efforts, the only currently approved clinically actionable biomarkers or companion diagnostics (CDx) predicting responses to ICI therapies are still limited to PD-L1 immunohistochemistry, tumor mutational burden (TMB), and microsatellite instability high (MSI-H)/mismatch repair deficient (dMMR) tumor profiling [25–28]. These biomarkers unfortunately only capture a single feature of the many possible factors contributing to ICI response or resistance and are independently too variable across both patients and lesions to be considered dependable enough for their independent mainstay. The integration of biomarkers may thus represent a better strategy suited to correctly predicting responses, and previously reported gene expression signatures associated with responses may also be used to add critical information. The development of a platform that is capable of scoring the performance of current and future experimental biomarkers, signatures or features in development against and across multiple datasets and tumor types, may reveal one or a combination of these that already works well enough, and it may also vastly improve our fundamental understanding of the key features of the design or pruning of robust predictive signatures of response for a panoply of different pathologies or treatments.

Founded in 2017, the SITC powered TimIOs initiative was created to facilitate cross-institutional collaboration in the building of a research platform aimed at addressing the critical needs of understanding tumor heterogeneity and identifying the fundamental differences between responders and non-responders, though the assistance of key stakeholders including technology providers, pharmaceutical companies, patients, clinicians, and academia. Derived from the Greek word for honest, TimIOs was designed to perform the role of the broker for the acquisition, pooling, and harmonization of underpowered pan-cancer transcriptomic and clinical ICI trial datasets, towards increasing patient numbers to gauge the performance of previously published gene signatures and immunogenomics features (IGFs).

Launched to accompany The Cancer Genome Atlas (TCGA) PanCancer Atlas [29] the Cancer Research Institute (CRI) iAtlas is an interactive web platform providing a set of analytical tools for explorations of tumor and immune cell interactions within the TME [30]. These tools allow researchers to explore and visualize associations between a variety of genomic characterizations of immune response, clinical phenotypes, germline genetics, and response to immunotherapy. To accelerate discovery to improve patient outcomes, the ICI analysis modules use direct comparison and multivariable statistical models to allow interactive explorations of the relationships or correlations between possible biomarkers of immune response and patient outcomes.

Towards the creation of a standardized bioinformatics workflow for evaluation of IGFs reported to associate with clinical responses to ICI therapies, the selection criteria of included datasets should be datasets from pan-cancer PD-1, PD-L1 or CTLA-4 inhibitor treated patients providing clinical metadata mapped to available raw transcriptomic data files, while inclusion of TCGA datasets provide prognostic estimates independent of immunotherapy treatment assisting the contrasting of IGFs that are actually ICI predictive from those that are merely prognostic and describing a patient population that may benefit from none or any and all applied treatment based on their inherent states of immunity. Published IGFs retained for testing should be those proposed to provide predictive estimates of response to ICI, or prognostic estimates from TCGA analyses, and these range from the expression of single genes, to classical TME gene expression signatures, and to others established as predictive ICI response signatures and/or scoring systems. By comparing selected IGFs across intersecting hazard ratios from ICI trials and TCGA data, those carrying significant associations with survival in both ICI and TCGA datasets elucidate prognostic biomarkers, while those that are significantly associated with ICI but not TCGA datasets are poised to elucidate truly predictive biomarkers serving as valid targets for the development of future CDx. With the validation of these previously proposed predictors of response to ICI will come the identification of what specific biological mechanism are behind these functional signatures, how far down these IGFs can be pruned without reducing their predictive power, and which current and/or emerging technologies will be best suited for their routine and reproducible profiling in patients.

### Best of SITC 2022 for clinical development and trials

Vidutolimod is a CpG-A toll-like receptor 9 agonist delivered in a virus-like particle. In a phase II trial, neoadjuvant vidutolimod plus nivolumab were evaluated in 30 patients with high-risk stage III resectable melanoma [31]. The combination was administered over 7 weeks presurgery and was continued for 48 weeks after surgery. Treatment was well tolerated with no dose-limiting toxicities. Promising clinical activity was shown with a pathological complete response (pCR) rate of 47% and a major pathological response (MPR) rate of 57%. MPR was associated with increased plasmacytoid dendritic cells and immune infiltrate.

Lifileucel is a one-time autologous TIL therapy that was investigated in 153 patients with advanced melanoma that had progressed after immune checkpoint inhibitor therapy [32]. ORR was 31% with eight complete and 40 partial responses. Median duration of response (DOR) was not reached at a median follow-up of 28 months, with 42% of the responses maintained for at least 18 months. Median overall survival (OS) was 13.9 months and median progression-free survival (PFS) was 4.1 months. Elevated lactate dehydrogenase and target lesion sum of longest diameters above the median were independently correlated with ORR. These findings of durable responses together with a favorable safety profile data support further investigation of lifileucel in heavily pretreated patients with advanced melanoma and high tumor burden.

Cabozantinib, a multiple receptor tyrosine kinase inhibitor, is being evaluated in combination with atezolizumab in advanced solid tumors in the COSMIC-021 phase Ib study [33]. In the cohort of 30 patients with advanced head and neck cancer pretreated with platinum-based chemotherapy, the combination showed moderate clinical activity with an ORR of 17% and disease control rate (DCR) of 60%. Response was comparable across different PD-L1 subgroups. Toxicity was manageable, with grade 3/4 treatment-related adverse events occurring in 47% of patients.

In a phase I trial, NeoTCR-P1, a personalized autologous T cell therapy for treatment of patients with solid tumors, was evaluated alone or in combination with IL-2 in 16 patients with solid tumors [34]. Neoantigen-specific T cell receptors (neoTCRs) were isolated from the patients’ circulating CD8 T cells, followed by non-viral precision genome engineering into an autologous apheresis product for infusion back into the patient. Seventeen of 22 neoTCR T cells were detected in post-infusion biopsies with 12 neoTCRs among the top 4% of CDR3 sequences detected. The targeted neoantigens were detected in 7 of 8 post-treatment biopsies (15 of 22 targets). These findings show the manufacturing feasibility and preliminary safety of NeoTCR-P1, along with T cell persistence and trafficking to different solid tumors.

CT-0508 is a cell product comprised of autologous monocyte-derived proinflammatory macrophages expressing an anti-human epidermal growth factor receptor (HER)2 CAR that is being investigated in a first-in-human clinical trial. CT-0508 product was manufactured from apheresis material collected from patients enrolled in the CT-0508 phase 1 clinical trial [35]. CT-0508 was successfully manufactured with high viability, purity and CAR expression. CT-0508 products showed a M1 macrophage phenotype and enhanced killing and phagocytosis of HER2-expressing tumor cells over autologous untransduced macrophages. CAR-antigen interaction drove cell product activation and amplified the M1 polarization status of CT-0508 CAR-M.

Eftilagimod-α is a soluble LAG-3 protein that binds to a subset of major histocompatibility complex (MHC) class II molecules to mediate activation of antigen presenting cells and CD8 T-cells. TACTI-002 is a phase II study of eftilagimod-α in combination with pembrolizumab in patients not selected for PD-L1 expression with immunotherapy-naïve (first-line) and immunotherapy-refractory metastatic NSCLC and immunotherapy-naïve (second-line) patients with head and neck squamous cell carcinoma (HNSCC). In analysis of 88 patients, the most frequent adverse events were asthenia (28%), cough (27%), decreased appetite (22%), dyspnea (21%), fatigue (18%) and diarrhea (15%) [36]. Three patients discontinued due to treatment-related adverse events. ORR was 53% in 17 immunotherapy-naïve patients with NSCLC and 39% in 18 immunotherapy-naïve patients with HNSCC. Thus, the combination is safe and shows encouraging antitumor responses.

NT-I7 (efineptakin alfa) is a long-acting human IL-7 fusion protein that promotes T-cell development and has been shown to increase T-cell infiltration in combination with pembrolizumab. In a phase IIa study, patients with relapsed/refractory checkpoint inhibitor-naïve MSS-colorectal cancer and pancreatic cancer received NT-I7 every 6 weeks plus pembrolizumab every 3 weeks [37]. In this analysis, 53 patients were evaluable, of whom 74% had liver metastasis. ORR was 3.8% per Response Evaluation Criteria in Solid Tumors (RECIST) and 9.4% per immune-RECIST. Patients with non-liver metastasis had a 29% immune-ORR and 71% immune-DCR, while patients with liver metastasis had an immune-DCR of 26%. CD8 T-cell infiltration increased with treatment and was associated with improved OS.

## Trends in Immunotherapy

### Using neoadjuvant trials to develop novel therapies and biomarkers of response

Modulation of the immune environment can optimise targeting of head and neck cancer. Immune biomarker modulation was observed in an phase Ib clinical trial of patients with head and neck cancer treated with neoadjuvant cetuximab, an epidermal growth factor receptor (EGFR)-specific antibody, plus CD137 agonist urelumab [38]. Myeloid-derived suppressor cells were elevated in non-responders, as were CTLA-4-negative T regulatory cells. EGFR-specific T cells predict tumor response, with induction of immunity associated with clinical benefit. Cetuximab reduces T cell receptor diversity and promoted expansion in TIL samples; however, the magnitude of clonal expansion in the top 20 T cell receptor clonotypes was significantly higher in responder TILs and peripheral blood mononuclear cells (PBMCs) [39].

Neoadjuvant therapy provides a brief, preoperative window of opportunity that can be used to identify biomarkers of response and individualize subsequent treatment. In an analysis of patients from three clinical trials, response to neoadjuvant therapy based on pathologic downstaging was associated with significantly better disease-free survival (DFS) and OS [40]. In the CheckMate 358 trial, neoadjuvant nivolumab was generally safe and induced pathologic regressions in patients with resectable human papillomavirus (HPV)-positive and HPV-negative HNSCC tumors [41]. However, the response rate to monotherapy remains low in HNSCC and there is a need to better understand the multifactorial and personalised mechanisms of resistance involved and to identify new targets that may sensitize tumours to combination therapies.

In a phase II study to assess the safety and tolerability of nivolumab administered alone or in combination with ipilimumab or relatlimab, alterations in T cell receptor clonotypes and changes in transcription profiles in CD8 T cells from blood and tumor following monotherapy and combination immune checkpoint blockade were analysed in treatment-naïve patients with HNSCC [42]. Based on single cell RNA sequencing data available from 25 accrued patients, CD8 T cells in the tumor increased after treatment with combined nivolumab and ipilimumab. Patients receiving nivolumab combined with relatlimab had a post-treatment increase in CD8 T cell receptor repertoire diversity and had more T cell receptor clones occupying 50% of the repertoire post-treatment compared to baseline. Further analysis is ongoing to investigate changes in transcriptional profiles of expanded clones in tumors across different treatment arms and by response and to correlate these data with clinical outcomes.

### Immunotherapy data in genito-urinary cancers: what’s new and what’s confirmed

Patients with metastatic urothelial cancer have been characterized for a long time of a poor prognosis and limited treatment options after first-line platinum-based chemotherapy. In recent years, immune-checkpoint inhibitors have been investigated in first-line therapy but their addition to chemotherapy has failed to show an OS benefit over chemotherapy alone [43, 44]. However, in the JAVELIN Bladder 100 trial, the addition of maintenance avelumab to best supportive care significantly prolonged OS with a 31% decrease in the risk of death as compared with best supportive care alone in patients with unresectable locally advanced or metastatic urothelial cancer who had not progressed on first-line platinum-based hemotherapy [45]. Of interest, OS and PFS were prolonged with avelumab irrespective of first-line chemotherapy regimen (gemcitabine plus cisplatin or carboplatin) or number of cycles. In longer-term follow-up, prolonged OS with the addition of avelumab was maintained irrespective of best response to first-line chemotherapy [46]. In patients with PD-L1-positive tumors, an increased OS was observed.

Also, in the adjuvant setting of high-risk muscle-invasive urothelial carcinoma, nivolumab increased median disease-free survival compared with placebo in the intention-to-treat population and in patients with a PD-L1 expression ≥ 1% [47].

Immunotherapy for urothelial cancer is also being investigated in combination with targeted agents. The antibody–drug conjugate enfortumab vedotin showed high ORR with rapid responses in combination with pembrolizumab in previously untreated cisplatin-ineligible patients with locally advanced or metastatic urothelial cancer [48].

In metastatic renal cell carcinoma (RCC), several anti-PD-1 based approaches have been investigated, either in combination with ipilimumab or with tyrosine kinase inhibitors, and several clinical trials have shown an OS benefit versus sunitinib. In CheckMate 214, nivolumab plus ipilimumab maintained a 5-year OS survival benefit over sunitinib in patients with intermediate/poor-risk RCC [49]. Further to this, in the COSMIC-313 trial, patients with intermediate or poor risk RCC who received cabozantinib in addition to nivolumab and ipilimumab had a 27% lower risk of disease progression or death compared to those who received nivolumab and ipilimumab but with an increased risk of adverse events [50]. No OS benefit has yet been shown. In the CheckMate 9ER trial, nivolumab plus cabozantinib had a significant PFS and OS benefit versus sunitinib in patients with previously untreated clear-cell, advanced RCC [51, 52]. Similar survival benefits over sunitinib were seen in the KEYNOTE 426 trial with pembrolizumab plus axitinib [53].

### Colorectal cancer

In patients with metastatic MSI-H/dMMR colorectal cancer who had not previously received treatment, treatment with pembrolizumab resulted in significantly longer PFS than chemotherapy, with a durable antitumor activity [54]. The study did not meet its OS endpoint (for the prespecified limit of significance); however, this result has a more formal than substantial value, also considering the crossover in the treatment [55]. In the SAMCO-PRODIGE 54 trial of second-line treatment in patients with metastatic MSI-H/dMMR colorectal cancer, avelumab improved PFS versus chemotherapy with or without targeted agents [56]. ORR and DCR were similar between treatment arms, but disease control was maintained over 18 months with avelumab in most patients. These data support the view that patients with MSI-H/dMMR colorectal cancer should be treated early with immune checkpoint inhibitors.

In the CheckMate 142 trial, nivolumab plus low-dose ipilimumab showed clinically meaningful efficacy characterized by an ORR of 65%, DCR of 81%, and durable responses after a median 50.9 months of follow-up in previously treated patients with MSI-H/dMMR colorectal cancer [57]. ORR benefit was observed in all evaluated subgroups, including by *BRAF* or *KRAS* mutation status, and was consistent with that in the overall population. Median PFS and OS were not reached. At 48 months, the OS rate was 71%, and the PFS rate was 53%. The safety profile was manageable with no new safety signals identified.

Earlier use in neoadjuvant and adjuvant settings has also been investigated. The first neoadjuvant immunotherapy trial was NICHE, in which patients with dMMR and MMR-proficient (pMMR) colon cancer received nivolumab plus ipilimumab [58]. A major pathologic response (< 10% viable tumor rest) was shown in 31/32 dMMR tumors but only in 7 of 31 patents with pMMR. In NICHE-2, 106 of 107 (99%) patients with dMMR colon cancer had a pathologic response [59]. Similarly, treatment with the anti-PD-1 dostarlimab in 14 patients with dMMR rectal cancer resulted in a 100% complete clinical response rate after follow-up of 6 to 25 months [60].

However, high MSI represents only around 5% of colorectal cancers and there is a need for novel approaches for many patients with pMMR or MSS/MSI-low tumors. In one trial in patients with pMMR colorectal cancer, mutations in the polymerase epsilon (POLE) gene that generate proofreading defects led to higher TMB with more TILs and predicted anti-PD-1 efficacy [61]. POLE proofreading deficiency could be combined with TMB as a biomarker for cancer immunotherapy.

Patients without pathogenic POLE mutations need other strategies that can change immunologically ‘cold’ tumors into ‘hot’ tumors. These include combined immune checkpoint inhibition, immunotherapy-based combinations with chemotherapy and targeted therapy (e.g., antiangiogenic agents), radiation therapy, vaccines, and intratumoral strategies such as oncolytic viruses and bispecific antibodies. The combination of botensilimab, an Fc-enhanced CTLA-4 inhibitor, plus the PD-1 inhibitor balstilimab may offer a novel regimen. In 41 patients with MSS disease, 24% had achieved an objective response and the DCR was 73%, with responses ongoing in eight of 10 patients after a median follow-up of 5.8 months [62]. In another trial, the multi-tyrosine kinase inhibitor regorafenib plus nivolumab had a manageable safety profile and encouraging antitumor activity in patients with gastric and colorectal cancer [63]. Multi-kinase inhibitors are synergistic with anti-PD-1 blockade in patients without liver metastases, but benefits appear modest. Another approach is priming with chemotherapy. A 2-month priming phase with the oral alkylating agent temozolomide, followed by combination immunotherapy with low-dose ipilimumab plus nivolumab was associated with a 36% 8-month PFS rate, median PFS of 7.1 months and median OS of 18.5 months in 33 pre-treated MSS patients with silencing methylations in the MGMT promoter who completed the priming phase and were free from progression (24% of 135 patients who received temozolomide); the remaining 102 patients had progressive disease or died during priming [64]. This chemoimmune sensitisation strategy is promising but needs further development.

### Translating novel immunotherapy combinations in gastrointestinal cancer

Cancer-associated fibroblasts are one of the most abundant cell types within the dense pancreatic ductal adenocarcinoma (PDAC) stroma and mediate communication with immune cells in the TME. In primary PDAC, cancer-associated fibroblasts express highly elevated levels of IL-6, which fuels PDAC differentiation, proliferation, and progression, and promotes differentiation of immunosuppressive populations, e.g., myeloid-derived suppressor cells, T helper 17 cells, dendritic cells, and macrophages.

Combination therapy strategies involving IL-6 blockade in combination with antibodies targeting inhibitory immune checkpoint receptors have also shown promise in pre-clinical studies. For example, dual IL-6 and PD-1/PD-L1 blockade inhibits tumor growth and enhances effector T cell infiltration in PDAC murine models [65]. The efficacy of IL-6 blockade has recently been extended to combinations involving CTLA-4 blockade. In these studies, efficacy is dependent on CXCR3 on T cells, with in vivo blockade of CXCR3 preventing orthotopic tumor regression in the presence of combined treatment [66]. The combination of IL-6 and PD-1 blockade is being investigated in the Winship 4463 (NCT04191421) phase Ib/II clinical trial of siltuximab, a chimeric monoclonal antibody which targets IL-6, and the PD-1 inhibitor spartalizumab.

Other pathways are also viable targets in context of the pancreatic cancer stroma. Namely, heat shock protein-90 (Hsp90) is a chaperone protein that drives inflammatory pathways and represents a novel stromal target. Combined therapy with the Hsp90 inhibitor XL888 and anti-PD-1 was effective in C57BL/6 mice bearing syngeneic subcutaneous or orthotopic PDAC tumors [67]. Tumors from mice treated with both XL888 and anti-PD-1 had decreased activation of cancer-associated fibroblasts, increased CD8 + and CD4 + T-cell infiltrates and a unique transcriptional profile characterised by upregulation of genes associated with immune response, such as chemokines.

**Fig. 2:**
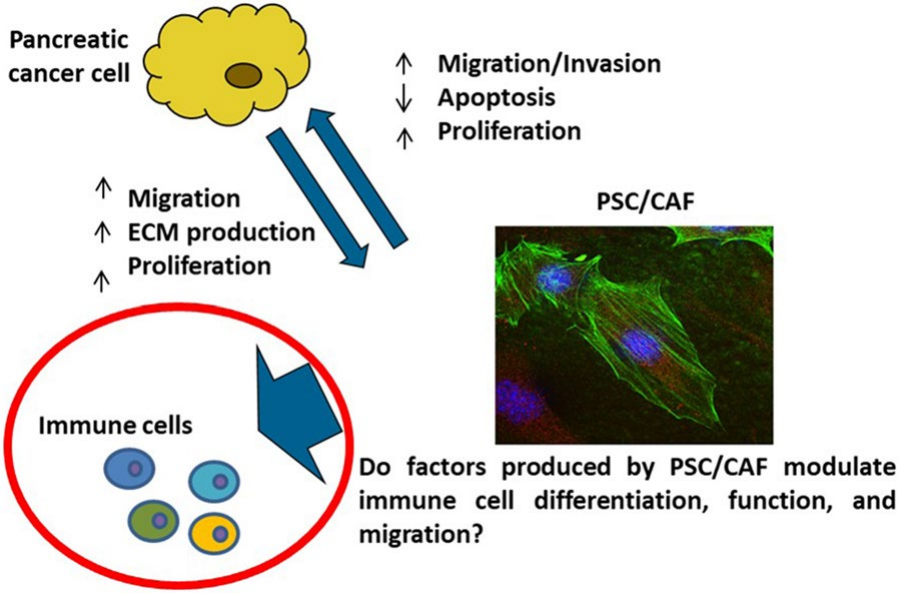
CAF-derived factors mediate communication with immune cells in tumor microenvironment

In biliary cancer, dual MEK/PD-L1 inhibition with atezolizumab plus cobimetinib improves PFS but with a low response rate and reduced T cell activation [68]. Immunotherapy combinations including cobimetinib plus atezolizumab warrant additional investigation and the combination of atezolizumab and the anti-CD27 antibody varlilumab is being assessed with or without the addition of cobimetinib in a randomized phase II study in previously treated unresectable biliary tract cancers (NCT04941287) (Fig. 2).

### Breast cancer immunotherapy: moving forward

Adding PD-1/PD-L1 inhibitors to standard chemotherapy enhances clinical efficacy in both metastatic and early-stage TNBC, although the efficacy of PD-1/PD-L1 blockade is dependent on PD-L1 expression only for patients with advanced, incurable TNBC. The randomized, placebo-controlled phase III IMpassion130 clinical trial demonstrated that patients with metastatic TNBC and PD-L1 immune cell-positive tumors derive clinical benefit from atezolizumab plus nab-paclitaxel compared with nab-paclitaxel with placebo [69]. Patients with metastatic PD-L1 + tumors and a richer tumor immune microenvironment were more likely to derive clinical benefit. Patients with metastatic PD-L1 + TNBC that was genomically unstable (homologous recombination deficiency and higher TMB), more prone to DNA damage repair, or with higher levels of cell cycle proliferation were also more likely to benefit from the addition of atezolizumab to nab-paclitaxel.

Paired tumors obtained at baseline, on treatment, and at disease progression were evaluated to interrogate the mechanisms of action and resistance with the use of atezolizumab combined with nab-paclitaxel. Immune phenotypes were assessed according to the geographic location of tumor stromal immune cells (inflamed, excluded or desert). TNBC molecular subtypes were assessed (basal-like immune-activated (BLIA), basal-like immune-suppressed (BLIS), luminal androgen receptor (LAR) and MES) by RNA sequencing [70]. PD-L1 immune cell-positivity in either primary or metastatic tumor tissue was linked to PFS and OS benefit with atezolizumab plus nab-paclitaxel. Whereas PFS benefit was associated with both the immune-inflamed and immune-excluded phenotypes, OS benefit was only associated with the immune-inflamed phenotype. PFS benefit was observed in patients with either the BLIA or BLIS molecular subtypes, and OS benefit was limited to the BLIA molecular subtype. Potential mechanisms of resistance were observed in PD-L1-positive subgroups, including the LAR molecular subtype, increased tumor angiogenesis, epithelial-mesenchymal transition, Hedgehog pathway, oestrogen response and tumor necrosis factor signalling pathways for PFS, and the BLIS and LAR molecular subtypes for OS. While analysis showed that tumor immune phenotypes were somewhat fluid and changed on treatment and at disease progression, the molecular subtypes were more stable even in the presence of atezolizumab. These findings are hypothesis-generating and require validation in an independent data set.

### Progress in immunotherapy for ovarian cancer

Although immunotherapy has proven successful in several solid tumors, the benefits achieved in ovarian cancer have been modest. However, there is evidence of immune recognition and potential for vaccine-based approaches. NY-ESO-1 may be a potential target for antigen-specific immunotherapy in ovarian cancer, with its expression predictive of an aggressive phenotype [71]. PD-1/PD-L1 inhibitors as single agents have poor activity in advanced ovarian cancer, with response rates of around 10%, which may be due to compensatory upregulation of inhibitory receptors. In a trial of combined immune checkpoint blockade, ipilimumab plus nivolumab improved the response rate compared with nivolumab alone in women with persistent or recurrent ovarian cancer [72]. PFS also improved, although was still limited. In other trials, neither avelumab plus pegylated liposomal doxorubicin nor avelumab alone significantly improved PFS or OS versus chemotherapy alone in patients with platinum-resistant ovarian cancer [73], while adding avelumab to a standard platinum-based chemotherapy regimen followed by avelumab maintenance versus chemotherapy alone did not improve PFS in patients with newly diagnosed advanced ovarian cancer [74]. Also, the addition of atezolizumab to platinum-based chemotherapy and bevacizumab in newly diagnosed stage III or IV ovarian cancer did not improve PFS or OS [75]. These and other studies confirm the lack of efficacy of immune checkpoint inhibitors as monotherapy or in combination in ovarian cancer. Biomarkers are needed to predict response to immune therapies, as are additional strategies to improve response. PD-L1 expression has poor predictive value for immunotherapy response in ovarian cancer and neither *BRCA1/2* mutations nor TMB appear to predict benefit. However, IFN transcriptional signature and tumor mutational signature 3 (homologous recombination deficiency) are positively associated with prolonged PFS with combined Poly(ADP-ribose) polymerase (PARP) and immune checkpoint inhibition [76].

IMC-F106C is the first T cell receptor bispecific protein targeting CD3 and PRAME, the most broadly expressed cancer testis antigen, which is homogenously expressed in multiple tumors. IMC-F106C was well tolerated and demonstrated durable RECIST partial responses and circulating tumor (ct)DNA response in PRAME-positive patients across multiple tumor types, including ovarian cancer [77].

The effectiveness of immunotherapies in ovarian cancer may be limited by the multiple immune suppressive networks in the TME. Effective immunotherapy in ovarian cancer will require a two-step approach to counteract innate and adaptive mechanisms of immune resistance and to generate an effective antitumor immune response, e.g., via vaccines or ACT.

### Trends in lymphoma immunotherapy and lessons for solid tumors

Effectively primed T cells can target large lymphoma tumors. Recent data have shown that epcoritamab, a CD3 x CD20 T cell engaging, bispecific antibody, can achieve durable responses in highly refractory patients with large B-cell lymphoma, including those previously treated with CAR T cell therapy. Similarly, another CD20 x CD3 T-cell-engaging, bispecific antibody, glofitamab, was effective in patients with relapsed or refractory diffuse large B-cell lymphoma [78]. In addition, CD19 CAR T therapy has also shown efficacy in earlier stage disease. However, effective priming of T cells is difficult with suboptimal tumor antigen presentation. Often, the antigens to target are unknown and can vary between individuals. Although having an immune inflamed tumor may correlate with response to immune checkpoint blockade, many patients with ‘hot’ tumors still fail to respond. This suggests a hot tumor phenotype is not the cause of response. Rather, antitumor T cell responses critically depend on antigen-presenting dendritic cells. Activation, loading and mobilisation of these cells offers a potential strategy to improve response to T cell-based therapies.

Priming antitumor CD8 + T cells requires cross-presentation. In situ vaccination combining Fms-like tyrosine kinase 3 (Flt3), poly-ICLC, and radiotherapy induced antitumor CD8 + T cell responses in patients with advanced stage indolent NHL, some of whom had a heavy tumor burden [79]. Systemic regression of 75% was observed in some patients, with responses often durable. Systemic (abscopal) cancer remission was also observed. Non-responding patients developed a population of PD1 + CD8 + T cells, and murine tumors became responsive to PD-1 blockade. Expansion of dendritic cells by Flt3L administration also induced parallel amplification of Newcastle Disease Virus- and tumor-specific T cells, including CD8 + T cells reactive to newly described neoepitopes, promoting long-term tumor control and indicating that mobilizing dendritic cells to increase tumor antigen cross-presentation can improve the efficacy of oncolytic virotherapy.

GS-3583, an Flt3 agonist Fc fusion protein can expand conventional dendritic cells. In patients with advanced solid tumors, GS-3583 was safe and well tolerated and induced dose-dependent expansion of conventional dendritic cells in a phase Ib trial, suggesting further investigation in combination with other agents is warranted [80].

## Drivers of immune responses

### Immunogenicity and therapeutic cross-protection to NRAS Q61 public neoantigens

Mutations that alter protein function to promote oncogenesis, so-called driver mutations, typically occur in tightly constrained hotspot regions and can systematically reappear across patients. A peptide containing a hotspot mutation bound by a relatively common HLA allele can result in a ‘public’ neoantigen that is shared across patients [81, 82].

Mutant NRAS causes therapeutic resistance to PD-1 and anti-EGFR blockade. Patients with melanomas harboring BRAF V600 or NRAS Q61 alterations have a shorter time to treatment failure than those with NF1 or other driver alterations [83]. An immuno-peptidomic screening approach revealed the existence of a family of NRAS public neoantigens resulting from all prevalent NRAS Q61 hotspot mutations. Each of the identified shared neopeptides was found to be spontaneously immunogenic in HLA-A*01-positive cancer patients. TCRs cloned from these patients were associated with high potency, as measured by the ability to lyse HLA-A*01 + -positive melanoma cell lines that naturally harbor NRAS mutations. NRAS public neoantigen-specific T cells were detected in both peripheral blood and TILs. NRAS public neoantigen-reactive T cells were also responsive to ipilimumab and nivolumab treatment and associated with objective tumor regression. A panel of NRAS TCRs were found to provide cross-protection to both NRAS Q61R and NRAS Q61K mutant peptides, providing the foundation for a new class of TCR therapeutics [84].

### Intracranial administration of immune checkpoint blockade in patients with recurrent glioblastoma

GlITIpNi is a multi-cohort adaptive phase I clinical trial that assessed the intracerebral administration of ipilimumab with or without nivolumab in combination with intravenous low-dose nivolumab in 27 patients with a resectable glioblastoma recurrence. Most patients were initially diagnosed with a WHO grade 4 glioblastoma and most had been treated at first diagnosis by surgery followed by adjuvant radiation therapy with concomitant and adjuvant temozolomide chemotherapy. In the first two cohorts (cohort 1, ipilimumab alone and cohort 2, ipilimumab plus nivolumab), treatment was safe and feasible, and associated with encouraging antitumor activity [85].

In subsequent cohort expansion, the safety of intratumoral ipilimumab plus nivolumab followed by repeated intracavitary or intrathecal admin of nivolumab with or without increasing doses of ipilimuamb was investigated in 32 patients [86]. A further cohort of 11 patients underwent intracranial administration of autologous myeloid dendritic cells (myDC) in combination with ipilimumab and nivolumab [87]. All patients received the planned pre-operative 10 mg intravenous nivolumab dose, underwent the planned neurosurgical procedure and perioperative administration of study treatment, and all patients in the relevant cohort underwent successful leukapheresis and myDC isolation.

Overall, intracranial administration of ipilimumab and nivolumab was feasible and associated with an acceptable safety profile. There was an unanticipated low incidence and low severity of immune-related adverse events and rapid clearance of nivolumab from the cerebrospinal fluid. Dose-limiting toxicity (neutrophilic pleocytosis) occurred with intracavitary ipilimumab 5–10 mg every 2 weeks. A potential beneficial effect on OS seemed to be limited to patients with resectable recurrences, with central nervous system tumor burden suspected to determine treatment outcome. There was no indication that repeated intracavitary administration of nivolumab and ipilimumab offered an additional benefit following pre-operative injection. There is potential for predictive and/or prognostic tissue biomarkers (including B7-H3) to guide patient selection and investigation of tumor-antigen specificity of lymphocytes present in the cerebrospinal fluid may provide opportunities for antigen-specific therapies.

**Fig. 3:**
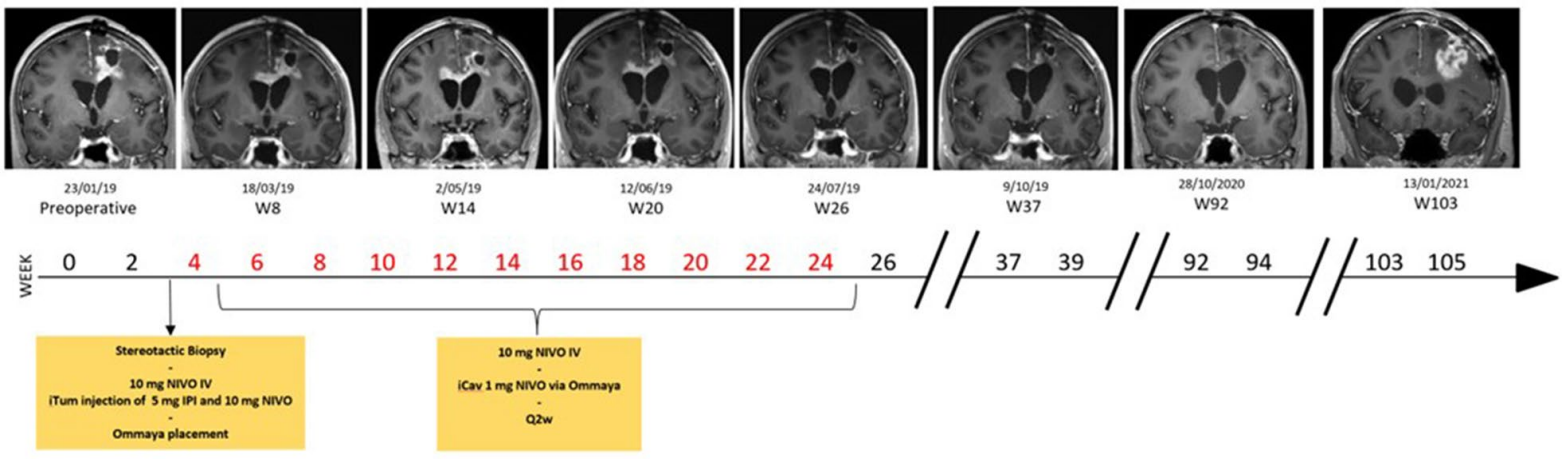
Case illustration. A 60-year-old female diagnosed with de novo glioblastoma (IDH1wt, MGMT promoter un-methylated) was diagnosed with progression of disease following concomitant RT/temozolomide and 4 adjuvant cycles of temozolomide. She was enrolled in Cohort-3 of the Glitpni trial. Initial progression on MRI of the brain (illustrated by representative coronal gadolinium enhanced T1 images) evolved to a complete response that persisted up to week 103

Intracranial ipilimumab plus nivolumab administration is under further investigation as a backbone for complementary repeated intra-cavitary administration of ipilimumab (dose-escalation) and complementary intracerebral administration of autologous myDCs (Fig. 3).

### Bispecific antibodies in cancer immunotherapy

Bispecific antibodies are a diverse family of antibodies or antibody constructs that recognize two epitopes or antigens. Most are bispecific T-cell-engagers (BiTEs), designed to redirect and/or activate CD3-expressing cytotoxic T cells against a specific tumor target on malignant cells. Others include therapies that target immune checkpoints, oncogenic signaling pathways and cytokines. Bifunctional fusion proteins are a subset that are typically devoid of an Fc region. T-cell bispecific antibodies (TCBs) elicit antitumor responses by cross-linking of T cells to target tumor cells. TCB-mediated polyclonal T cell activation is independent of the T cell receptor specificity and does not require costimulatory signals. Factors normally affecting the efficiency of checkpoint inhibitors to mount an endogenous antitumor immune response, including MHC downregulation, antigen presentation, the frequency of antigen-specific T cells, T cell receptor affinity, and T-cell avidity, are less relevant for TCB activity.

MEDI5752 is a PD-1/CTLA-4 bispecific antibody engineered to preferentially bind CTLA-4 in PD-1-positive activated T cells, thereby enhancing CTLA-4 blockade and yielding greater T cell proliferation than possible with anti-CTLA-4 doses that are tolerable in the clinic. MEDI5742 has shown encouraging antitumor activity across a range of immunotherapy-naïve solid cancers. In a randomized cohort in a phase Ib/II trial, 41 patients with NSCLC were administered four cycles of carboplatin plus pemetrexed followed by maintenance therapy with pemetrexed plus either 1500 mg of MEDI5752 every 3 weeks or pembrolizumab. Subsequently, 50 patients in a single arm-cohort received 750 mg of MEDI5752 every 3 weeks in combination with carboplatin plus pemetrexed [88]. In the randomised cohort, MEDI155752 plus carboplatin improved duration of response, PFS and OS compared with chemotherapy. Lower-dose MEDI5752 also showed encouraging antitumor activity, especially in patients with PD-L1 < 1% expression, with improved tolerability.

Another bispecific antibody in development is vudalimab (XmAb20717), which simultaneously targets PD-1 and CTLA-4 and binds preferentially to PD-1/CTLA-4 dual-positive cells. Vudalimab monotherapy was generally well-tolerated and associated with complete and partial responses in patients with multiple tumor types. In a phase II study, vudalimab is being assessed as monotherapy or in combination with other anticancer agents in patients with metastatic castration-resistant prostate cancer (mCRPC) that progressed following treatment with ≥ 2 lines of anticancer therapy [89]. Patients with mCRPC will be enrolled into the following parallel cohorts based on the presence or absence of molecular abnormalities from prior sequencing of metastatic tumor: aggressive variant (cohort 1), homologous recombination deficient or CDK12 mutation positive PARP inhibitor progressor (cohort 2) or PARP inhibitor-naïve (cohort 3), MSI-H or dMMR (cohort 4), and no targetable mutation (cohort 5). All patients will receive vudalimab 10 mg/kg every 2 weeks. Cohorts 1, 2, and 5 will also receive carboplatin plus cabazitaxel (or docetaxel) while cohort 3 will also receive olaparib.

Bispecific T cell engaging receptor (TCER®) molecules consist of an affinity maturated TCR, a humanised T cell-recruiting antibody and an Fc-part conferring extended half-life and favorable stability characteristics. IMA402 targets an HLA-A*02-presented peptide derived from PRAME, which is highly prevalent across multiple solid tumors. In preclinical xenograft mouse models, IMA402 led to consistent tumor regression including complete remissions and showed a serum half-life of several days [90]. IMC-F106C is the first TCR bispecific protein targeting CD3 and PRAME and was well tolerated and showed durable RECIST partial responses and ctDNA response in PRAME-positive patients across multiple cancer types [77].

### Harnessing novel CD4 T cell-based therapies against solid tumors

CD26 is a surface glycoprotein that distinguishes three human CD4 + T cell subsets with varying degrees of responsiveness to tumors: one with regulatory properties (CD26^neg^), one with a naïve/central memory phenotype (CD26^int^), and one with a durable stem memory profile (CD26^high^) [91]. CD26^high^ T cells secreted T helper cell 17 (Th17) cytokines, including IL-17A. CD26^high^ Th17 cells mediate robust tumor immunity. Single-cell sequencing reveals that CD26^high^ T cells are molecularly unique from Th17 cells. While both CD26^high^ and Th17 cells engineered to express mesothelin-specific CARs were therapeutic in murine models of mesothelioma, human CD26^high^ T cells mediated more durable immune responses and persisted relative to the other CD4 + T cell subsets (Th1, Th2, and Th17), with bulk CD4 or Th17 cell therapies producing only transient delays in tumor growth [92]. CD4 + CD26^high^ T cells did not require CD8 + CAR T cells for persistence in the tumor.

CD4 + CD26^high^ T cells redirected with CAR have enhanced functional and antitumor activity versus classic human subsets or unselected CD4 + T cells. Potent CD26 + Th17 therapy may have potential as next-generation ACT for non-responders to immune checkpoint blockade and is being investigated in patients with PDAC.

Recent work has now shown that CD26highT cells might be a marker of responsiveness to checkpoint blockade therapy. In fact, circulating CD4^+^CD26^high^ T cells were vastly reduced in melanoma patients compared to healthy subjects in a clinical analysis of patients in Naples, Italy. In addition, a significant association was observed between a low baseline percentage of CD4^+^CD26^high^ T cells and clinical outcomes, measured as overall survival and progression-free survival. As well, it was determined that patients with clinical benefit from nivolumab therapy had far more circulating CD4^+^CD26^high^ T cells than patients with non-clinical benefit at one year post therapy. Also, at this timepoint, pre-treatment proportion of circulating CD4^+^CD26^high^ T cells was correlated with Disease Control and best Overall Response Rate. Collectively, this work implies that the presence of CD26-expressing T cells in patients might be a marker of responsiveness to PD-1-based therapies. [93]

### Targeting neutrophils to enhance antitumor immunity

Neutrophils recruited to the TME can limit or worsen tumor progression. Multiple factors can modulate neutrophil function in the TME, and even within the same patient, neutrophils are exposed to different niches and cues within the TME that include products of damage (DAMPs), cytokines and chemokines, hypoxia, nutrients, and proximity to tumor cells and other immune cells. In addition, whether neutrophils in the TME are friends or foes can also depend on specific cancer therapies. In a meta-analysis of expression signatures from approximately 18,000 human tumors across 39 malignancies, intratumoral neutrophil signature had the strongest association with worse survival [94].

Using malignant effusions from patients with metastatic solid tumors as authentic components of the TME, we investigated how the TME reprograms neutrophils to acquire T cell suppressor function. In newly diagnosed epithelial ovarian cancer, we observed that ascites fluid contains morphologically mature neutrophils that are T cell suppressive [95]. Circulating neutrophils from OC patients were not intrinsically immunosuppressive, but acquired a suppressor phenotype, characterized by inhibition of CD3/CD28 stimulated T cell proliferation and activation following exposure to ovarian cancer ascites fluid supernatants (ASC [96]). This same suppressor phenotype was induced in normal donor neutrophils exposed to ASC. Neutrophil suppressors inhibited proliferation of naïve, central memory and effector memory T cells, and of tumor-associated lymphocytes from patients with newly diagnosed ovarian cancer [96, 97]. The induction of neutrophil suppressor function required a number of pathways, including complement signaling and NADPH oxidase [96, 97]. A similar complement-dependent neutrophil suppressor phenotype was induced by malignant effusions from patients with different metastatic cancers, demonstrating the generalizability of our findings. We also observed that ASC augments neutrophil trogocytosis of T cell membranes, an effect that was partially dependent on the CD11b/CD18 integrin and expected to injure T cells [97]. In response to these signaling and injurious cues, we observed that T cells acquire an immunoparalysis, characterized by impaired NFAT translocation, IL-2 production, glucose uptake, mitochondrial function, and mTOR activation required for T cell activation and proliferation [97].

Several studies in tumor-bearing mice point to complement signaling promoting tumor growth and obstructing antitumor immunity in part through recruitment of neutrophils/polymorphonuclear myeloid-derived suppressor cells to the TME [98, 99]. Based on these results and our work, we are undertaking a clinical study of complement C3 inhibition combined with checkpoint blockade in patients with recurrent metastatic OC and persistent malignant effusions (NCT04919629).

### Genomic profiling of the T cell regulome in adjuvant immune checkpoint inhibition in melanoma

There is an urgent clinical need to identify reliable biomarkers of immune checkpoint inhibition in melanoma. Current predictors are mainly centred on the TME and do not sufficiently explain the heterogeneity of outcomes observed for immune checkpoint inhibitor-treated patients. Baseline markers of host immunity are relatively under-investigated. Recent evidence points to cytotoxic T cells, including CD4 + and CD8 + T cells, as important determinants of host immunity. Peripheral T cell activation/differentiation is governed by transcriptional regulatory networks that determine T cell fates by altering regulome sites (enhancers, promoters, methylation sites, lncRNAs). We hypothesized that host baseline CD4 + /CD8 + regulome reprogramming affects outcomes with immune checkpoint inhibition, which could lead to the discovery of more personalised biomarkers and discovery of new targets.

Multi-omics analysis was performed to identify baseline peripheral biomarkers of adjuvant immune-checkpoint inhibition using bulk RNA-sequencing of CD8 + and CD4 + cell populations. Samples were from the phase III CheckMate-915 study (NCT03068455) that compared nivolumab and ipilimumab versus nivolumab alone in patients who underwent complete surgical removal of stage III-IV melanoma. Baseline analysis showed differential gene expression in peripheral CD4 + T-cells between patients who relapsed after adjuvant nivolumab versus those who did not. There was significant enrichment of immune-related pathways with both single-agent nivolumab and the combination regimen at baseline CD4 + T cells. Expression of IGHD in both CD8 + and CD4 + T cells was associated with response to adjuvant nivolumab, while low ELF3 expression was significantly associated with worse recurrence-free survival (RFS). ELF3 is a pro-inflammatory transcription factor that stimulates HLA-C expression and is a prognostic factor in several cancers. Low GAREM1 expression was significantly associated with improved RFS, supported by significantly elevated chromatin openness in GAREM1 nivolumab-sensitive patients. GAREM1 plays both stimulatory and inhibitory roles in T cell development and maturation (including CD4 and CD8 cells) and promotes activation of the MAPK/ERK signalling pathway in T cells. There was supporting evidence for an association with a new genomic marker, HMAX, with resistance to both single-line nivolumab as well as combination therapy in CD8 and CD4 T cells. HMAX has a role in apoptosis and cell proliferation (including T cells) and is a potential novel biomarker of immune checkpoint inhibition in metastatic melanoma. Validation of these potential biomarkers is ongoing, with pre/post treatment and functional analyses underway.

### Mechanisms of immune checkpoint inhibitor toxicities across tumor types

Immune-related adverse events (irAEs) are driven by numerous potential mechanisms, but most essentially leads to a loss of tolerance. Mitigation strategies to manage irAEs, such as steroids, are crude and there is mixed data regarding the issue of whether the treatment of irAEs reduces the benefits of immunotherapy.

While some studies have suggested that the use of steroids to treat ipilimumab toxicity does not seem to be associated with reduced benefit, one sobering report showed that higher steroid dosing for ipilimumab-induced hypophysitis is associated with worse survival in patients with melanoma that in those who received replacement dose steroids [100]. Furthermore, baseline use of steroids has also been associated with worse outcomes. Specifically, in patients with NSCLC who were treated with PD-1/PD-L1 blockade, those on baseline steroids of greater than 10 mg of prednisone (or its equivalent) had decreased ORR, PFS and OS [101]. Finally, early use of high-dose steroids (within 8 weeks of starting PD-1 inhibitor monotherapy) for irAEs was associated with poorer PFS and OS in patients with melanoma [102].

Still, as steroids are the mainstay of irAEs, they need to be used in the right way for the right patient. In a retrospective analysis of anti-PD-1-treated patients with physician assessed, treatment-related diarrhea, 30% of those who underwent endoscopy did not have enterocolitis [103]. In those who had biopsy-confirmed enterocolitis, approximately a third had microscopic colitis (e.g., not seen on endoscopic exam but confirmed on biopsy) and two thirds had macroscopic colitis. In those with microscopic colitis, systemic glucocorticoid use as well as second line immune suppression with tumor necrosis factor (TNF) inhibition was lower, as oral budesonide was effective at controlling symptoms in most patients. Additionally, these patients were more likely to continue immune checkpoint inhibitor therapy than those with macroscopic colitis. In another study in patients with immune checkpoint inhibitor-induced nephritis, rapid tapering of steroids over 3 weeks resulted in similar renal outcomes to standard of care longer duration of steroid use [104].

An alternative approach to steroid use is to target mechanisms of toxicity that are separate from mechanisms of antitumor immunity. In immune-related colitis, there is increased frequency of cytotoxic and cycling T cell clusters, and tissue-resident memory T cell clusters are reduced [105]. There are shared T cell receptors between tissue-resident memory and colitis-associated cytotoxic/cycling T cell clusters. Gene expression analysis suggests potential targets distinct from antitumor immunity (i.e., TNFR1 expression). In immune-related myocarditis, increased intramyocardial T cells and the upregulation of multiple inflammatory pathways, in particular IFN responses, were observed across multiple immune and non-immune cell types [106]. These pathways could be therapeutic targets to manage immune-related myocarditis.

### Long-term toxicity and the impact on quality of life

Immune checkpoint inhibition can provide long-lasting durable responses in patients with metastatic disease. However, while acute toxicities of immune checkpoint inhibitors have been well characterised, there has been less focus on the development of chronic toxicities. These may be more frequent than initially believed, with data indicating that over 40% of patients may experience chronic side effects after anti-PD-1/PD-L1 therapy [107]. Although often low-grade, these persistent effects can affect the endocrine, rheumatological, pulmonary, neurological and other organ systems.

Although acute toxicities often resolve with steroid therapy, this is not always the case and some may develop into a chronic phenotype. Immune-related adverse events may fail to resolve because of smouldering inflammation or burnout. In smouldering inflammation, immune checkpoint inhibitors induce persistent subacute or chronic inflammation that resembles classic autoimmunity, e.g., inflammatory arthritis. Burnout refers to irreversible damage of the relevant cells, preventing physiological recovery, such as with endocrinopathies in which hormone-secreting cells are irretrievably damaged by the inflammatory process.

**Fig. 4:**
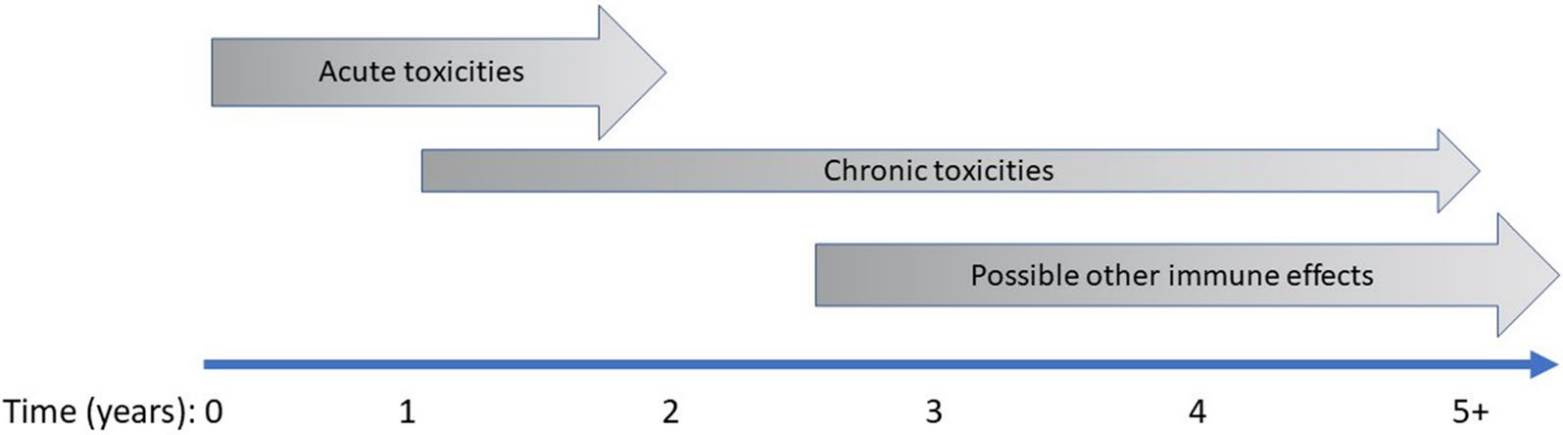
Long-term toxicity

Chronic toxicities have a possible impact on other immune processes, with perturbations caused by immune checkpoint inhibitors having a long-term influence on diverse pathobiological processes including atherosclerosis, obesity and neuroinflammation. For example, inactivation of PD-1/PD-L1 potentiates promotes the infiltration of macrophages, CD4 + T cells and CD8 + T cells into atherosclerotic plaques, suggesting that PD-1 checkpoint inhibitors could potentially increase the atherosclerosis burden and/or plaque rupture. This is supported by clinical data that indicated a three-fold higher incidence of atherosclerotic cardiovascular events in the 2 years following immune checkpoint inhibitor treatment compared with the 2 years preceding treatment [108]. In contrast to this, a retrospective, cohort study of patients with melanoma treated with immune checkpoint inhibitor therapy did not show any increase in liver inflammation post-treatment [109]. Preclinical studies also suggest that adipose tissue T cells express markers of T cell exhaustion and that T cells have a key role in obesity-related complications, such as steatohepatitis. Adipocyte-dependent PD-L1 knockout resulted in decreased tumour growth but increased obesity-associated inflammation in mice [110]. However, follow-up of over 200 patients treated with anti-PD-1/PD-L1 inhibitors did not show any increase in body mass index after 2 years, although there was a slight increase in adiposity and skeletal muscle mass [111]. The effects of immune-checkpoint blockade on a diverse range of immune processes are an important of research for cancer survivors (Fig. 4).

### Evidence for adapting radiotherapy to immune checkpoint blockade

In preclinical studies, radiation has been shown to be a powerful partner to immune check point blockade. Radiation-induced cytosolic DNA activates cGAS/STING inducing interferon production with recruitment and maturation of dendritic cells., In parallel, as part of DNA damage response transcription of mutated genes in the tumor cells can elicit presentation of neopitopes that contribute to an adaptive immune response with the generation of antitumor T cells. This ability of radiation to activate both the innate and adaptive immune system in the irradiated tumor mimics the immune response to a viral infection. Several challenges to translating preclinical work to the clinic, include the complexity and diversity of human immune responses to cancer, the fact that radiotherapy also elicits immunosuppressive effects y, and the evidence that the type of sequencing of radiation with immunotherapy may affect the success of this combination. These reasons are gradually emerging to explain the failure of some clinical trials of radiation and immunotherapy. For instance, in the JAVELIN trial, avelumab plus standard-of-care chemoradiotherapy failed to improve PFS and OS when compared to chemoradiotherapy alone in patients with locally advanced squamous cell carcinoma of the head and neck [112]. In this trial, use of standard radiation doses and treatment fields (inclusive of neck nodes) likely abrogated the immune fitness of patients. Preclinical data shows that radiation ablation or surgical removal of draining nodes eliminate the tumor response to immune checkpoint blockade, including loss of type I dendritic cells and type I IFN signaling [113]. Evidence for preserving immune fitness when treating with radiation and immunotherapy also emerged from a recent phase II trial comparing neoadjuvant durvalumab alone with neoadjuvant durvalumab plus stereotactic radiotherapy in patients with NSCLC. With comparable tolerability in both arms, MPR rate was significantly increased with the addition of radiotherapy (53% vs 7%); this was increased even more in EGFR-negative patients [114]. Importantly, the trial required radiotherapy to primary lung cancer only, excluding draining nodes even among the 13 patients with positive nodes before surgery. Durvalumab plus radiation to the primary tumor increased MPR in the nodes in 4/6 of these patients compared to 1/6 treated by durvalumab only, confirming the relevance of excluding draining nodes when radiation is used with immune checkpoint inhibition.

However, radiotherapy also has ‘out of field’ effects, mediated by normal tissue exposure to low dose of radiation. In an NSCLC model, the radiotherapy induced secretome of activated lung-resident Scgb1a1 + club cells, outside the field of radiation, inhibited immunosuppressive myeloid cells, reduced pro-tumor inflammation, and enhanced the efficacy of immune checkpoint inhibition [115].

Sequencing of radiation therapy and immunotherapy can influence response. PD-1 blockade after irradiation can result in the expansion of polyfunctional intratumoral CD8 + T cells and induction of potent abscopal responses [116]. However, in the same model, administration of anti-PD-1 therapy before irradiation nearly completely abrogated systemic immunity, which is associated with increased radiosensitivity and death of CD8 + T cells. The subsequent reduction of effector CD8 + T cells at the irradiated tumor site generates a suboptimal systemic antitumor response and the loss of abscopal responses.

In conclusion, it seems advisable before designing and conducting new trials of radiotherapy combined with immunotherapy, to focus on better understanding the immune effects of radiation in irradiated patients. For example, in a study of patients with rectal cancer, immune gene expression profiling before preoperative radiotherapy identified differences between poor and good responders [117] providing a rationale for future studies.

### Enhancing clinical genomics datasets with multimodal electronic health record data and imaging

The widespread accessibility of extensive genomic datasets, such as those demonstrated by the Genomics Evidence Neoplasia Information Exchange (GENIE), has revolutionized the personalization of treatment approaches in oncology. The GENIE pan-cancer registry features data from over 110,000 tumors and 100,000 patients, drawn from various cancer centers in the US, Canada, and Europe (https://doi.org/10.1158/2159-8290.CD-21-1547).

In addition to genomics, large quantities electronic health records (EHR) are also becoming widely available. Providence Genomics, for example, has a cloud hosted data waewhouse with access to 1.8 million Providence patients, with cancer registries with 800,000 patients, and clinical pathway decision support for over 7000 patients. These EHR based real world data can be enriched by incorporating layers of ancillary data, such as high-resolution digitized images. For example, a dataset of microscopy images from breast biopsy specimens at the Providence Cancer Institute, which associates 175,000 biopsy slides from 11,000 unique patients with cancer registry, metastasis and mortality data [118]. Proteomics, metabolomics, spatial biology, and transcriptomics data can also be utilized to enrich these real-world datasets. In this context, Artificial Intelligence (AI) and machine learning present immense opportunities to analyze these datasets for biomarker discovery, clinical trial simulations, pathomics, and clinical trial enrolment. A key application of AI is in natural language processing, enabling scalable EHR processing. An example of this approach is the utilization of natural language processing models to extract outcomes from imaging reports and oncologist notes, linking them to genomic datasets, in a structured and reproducible manner [119], trained, tested and validated against a supervised manual medical record review performed according to the PRISSMM (Pathology, Radiology/Imaging, Signs/Symptoms, Medical oncologist assessment, and bioMarkers) framework.

Deep learning algorithms have proven particularly useful in histopathology, performing on par with trained pathologists for tumor detection and grading. These models are also used for mutation prediction, response prediction, and survival prediction. For instance, machine learning-based assessments of Tumor-infiltrating lymphocyte (TIL) levels in standard histologic images have shown correlation with responses to anti-PD-1 therapy in patients with NSCLC [120].

In conclusion, while immunotherapy advancements have significantly enhanced treatment options and outcomes for cancer patients, there is still a need for more effective strategies. Novel therapies are being explored, often as part of combination regimens. Therapies that promote a more immunologically active TME are of particular interest, especially when combined with immune checkpoint blockade. Identifying biomarkers for increased personalization of immunotherapy is also a focus, given the evolving complexity of the treatment landscape and the increasing therapeutic options for many patients.

The applications of recently improved Large Language Models such as those that underly technologies such as ChatGPT (GPT-3 and now GPT-4, 10. 1056/NEJMsr2214184) in this context could be manifold. They can potentially help in understanding and processing unstructured EHR data, identifying patterns and correlations within complex multimodal datasets, enhancing the capabilities of conventional natural language processing for information extraction, and potentially serving as a tool for hypothesis generation in the realm of biomarker discovery and personalized therapy.

## Conclusions

Advances in immunotherapy have significantly improved treatment options and outcomes for patients with cancer, and ongoing research in this field is likely to lead to even more effective therapies in the future. Patients who previously had limited treatment options are now benefiting from novel immunotherapeutic approaches that provide long-term responses and improved survival. However, many patients still fail to respond to immunotherapy and there remains a need for new treatment strategies.

Various approaches are being investigated, including novel treatments used both as monotherapy and, more typically, as part of combination regimens. In particular, therapies that promote an more immunologically active TME are a primary focus, especially when combined with immune checkpoint blockade. The identification of biomarkers and the increased personalization of immunotherapy is also an area of focus, especially given the more complex treatment landscape that is emerging and the availability of increased therapeutic options for many patients.

## Data Availability

Not applicable.

## Abbreviations

ACT: Adoptive cell transfer
AI: Artificial intelligence
ASC: Ascites fluid supernatants
BiTE: Bispecific T-cell-engagers
CAR: Chimeric antigen receptor
CDx: Companion diagnostics
CRS: Cytokine release syndrome
ctDNA: Circulating tumor DNA
CTLA-4: Cytotoxic T-lymphocyte-associated antigen-4
DCR: Disease control rate
DFS: Disease-free survival
dMMR: Mismatch repair-deficient
DOR: Duration of response
EGFR: Epidermal growth factor receptor
HER: Electronic health record
Flt3L: Fms-like tyrosine kinase 3 ligand
GENIE: Genomics Evidence Neoplasia Information Exchange
GvHD: Graft-versus-host disease
HER: Human epidermal growth factor receptor
HLA: Human leukocyte antigen
HNSCC: Head and neck squamous cell carcinoma
HPV: Human papillomavirus
Hsp: Heat shock protein
ICI: Immune checkpoint inhibitors
IFN: Interferon
IGF: Immunogenomics features
IL: Interleukin
irAEs: Immune-related adverse events
mCRPC: Metastatic castration-resistant prostate cancer
MHC: Major histocompatibility complex
MPR: Major pathological response
MSI: Microsatellite instability
MSS: Microsatellite stable
myDC: Myeloid dendritic cells
neoTCR: Neoantigen-specific T cell receptor
NHL: Non-Hodgkin lymphoma
NK: Natural killer
NSCLC: Non-small cell lung cancer
ORR: Objective response rate
OS: Overall survival
PARP: Poly(ADP-ribose) polymerase
PBMC: Peripheral blood mononuclear cell
pCR: Pathological complete response
PDAC: Pancreatic ductal adenocarcinoma
PD-1: Programmed death-1
PD-L1: Programmed death ligand-1
PFS: Progression-free survival
pMMR: Mismatch repair -proficient
POLE: Polymerase epsilon
RCC: Renal cell carcinoma
RECIST: Response Evaluation Criteria in Solid Tumors
RFS: Recurrence-free survival
TCGA: The Cancer Genome Atlas
TCB: T-cell bispecific antibody
TCR: T cell receptor
TGF: Transforming growth factor
Th: T helper
TIL: Tumor-infiltrating lymphocyte
TMB: Tumor mutational burden
TME: Tumor microenvironment
TNBC: Triple-negative breast cancer
TNF: Tumor necrosis factor

## References

[1] Rosenberg SA, Yang JC, Sherry RM, Kammula US, Hughes MS, Phan GQ (2011). Durable complete responses in heavily pretreated patients with metastatic melanoma using T-cell transfer immunotherapy. Clin Cancer Res.

[2] Robbins PF, Dudley ME, Wunderlich J, El-Gamil M, Li YF, Zhou J (2004). Cutting edge: persistence of transferred lymphocyte clonotypes correlates with cancer regression in patients receiving cell transfer therapy. J Immunol.

[3] Powell DJ, Dudley ME, Robbins PF, Rosenberg SA (2005). Transition of late-stage effector T cells to CD27+ CD28+ tumor-reactive effector memory T cells in humans after adoptive cell transfer therapy. Blood.

[4] Krishna S, Lowery FJ, Copeland AR, Bahadiroglu E, Mukherjee R, Jia L (2020). Stem-like CD8 T cells mediate response of adoptive cell immunotherapy against human cancer. Science.

[5] Parkhurst MR, Yang JC, Langan RC, Dudley ME, Nathan DA, Feldman SA (2011). T cells targeting carcinoembryonic antigen can mediate regression of metastatic colorectal cancer but induce severe transient colitis. Mol Ther.

[6] Liu E, Marin D, Banerjee P, Macapinlac HA, Thompson P, Basar R (2020). Use of CAR-transduced natural killer cells in CD19-positive lymphoid tumors. N Engl J Med.

[7] Hamieh M, Dobrin A, Cabriolu A, van der Stegen SJC, Giavridis T (2019). CAR T cell trogocytosis and cooperative killing regulate tumour antigen escape. Nature.

[8] Li Y, Basar R, Wang G, Liu E, Moyes JS, Li L (2022). KIR-based inhibitory CARs overcome CAR-NK cell trogocytosis-mediated fratricide and tumor escape. Nat Med.

[9] Kerbauy LN, Marin ND, Kaplan M, Banerjee PP, Berrien-Elliott MM, Becker-Hapak M (2021). Combining AFM13, a bispecific CD30/CD16 antibody, with cytokine-activated blood and cord blood-derived NK cells facilitates CAR-like responses against CD30+ malignancies. Clin Cancer Res.

[10] Nieto Y, Banerjee P, Kaur I, Bassett R, Kerbauy L, Basar R (2022). Innate cell engager (ICE®) AFM13 combined with preactivated and expanded cord blood (CB)-derived NK cells for patients with refractory/relapsed CD30+ lymphoma. Cancer Res.

[11] Shaim H, Shanley M, Basar R, Daher M, Gumin J, Zamler DB (2021). Targeting the αv integrin/TGF-β axis improves natural killer cell function against glioblastoma stem cells. J Clin Invest.

[12] Singh N, Lee YG, Shestova O, Ravikumar P, Hayer KE, Hong SJ (2020). Impaired death receptor signaling in leukemia causes antigen-independent resistance by inducing CAR T-cell dysfunction. Cancer Discov.

[13] Jain MD, Ziccheddu B, Coughlin CA, Faramand R, Griswold AJ, Reid KM (2022). Whole-genome sequencing reveals complex genomic features underlying anti-CD19 CAR T-cell treatment failures in lymphoma. Blood.

[14] Orlando EJ, Han X, Tribouley C, Wood PA, Leary RJ, Riester M (2018). Genetic mechanisms of target antigen loss in CAR19 therapy of acute lymphoblastic leukemia. Nat Med.

[15] Sotillo E, Barrett DM, Black KL, Bagashev A, Oldridge D, Wu G (2015). Convergence of acquired mutations and alternative splicing of CD19 enables resistance to CART-19 immunotherapy. Cancer Discov.

[16] Heard A, Landmann JH, Hansen AR, Papadopolou A, Hsu YS, Selli ME (2022). Antigen glycosylation regulates efficacy of CAR T cells targeting CD19. Nat Commun.

[17] Fraietta JA, Lacey SF, Orlando EJ, Pruteanu-Malinici I, Gohil M, Lundh S (2018). Determinants of response and resistance to CD19 chimeric antigen receptor (CAR) T cell therapy of chronic lymphocytic leukemia. Nat Med.

[18] Chen GM, Chen C, Das RK, Gao P, Chen CH, Bandyopadhyay S (2021). Integrative bulk and single-cell profiling of premanufacture T-cell populations reveals factors mediating long-term persistence of CAR T-cell therapy. Cancer Discov.

[19] Haradhvala NJ, Leick MB, Maurer K, Gohil SH, Larson RC, Yao N (2022). Distinct cellular dynamics associated with response to CAR-T therapy for refractory B cell lymphoma. Nat Med.

[20] Good Z, Spiegel JY, Sahaf B, Malipatlolla MB, Ehlinger ZJ, Kurra S (2022). Post-infusion CAR TReg cells identify patients resistant to CD19-CAR therapy. Nat Med.

[21] Selli ME, Landmann J, Terekhova M, Lattin J, Heard A, Hsu YS (2023). Costimulatory domains direct distinct fates of CAR-driven T cell dysfunction. Blood.

[22] Good CR, Aznar MA, Kuramitsu S, Samareh P, Agarwal S, Donahue G (2021). An NK-like CAR T cell transition in CAR T cell dysfunction. Cell.

[23] Upadhaya S, Neftelinov ST, Hodge J, Campbell J (2022). Challenges and opportunities in the PD1/PDL1 inhibitor clinical trial landscape. Nat Rev Drug Discov.

[24] Litchfield K, Reading JL, Puttick C, Thakkar K, Abbosh C, Bentham R (2021). Meta-analysis of tumor- and T cell-intrinsic mechanisms of sensitization to checkpoint inhibition. Cell.

[25] Patel SP, Kurzrock R (2015). PD-L1 expression as a predictive biomarker in cancer immunotherapy. Mol Cancer Ther.

[26] Sul J, Blumenthal GM, Jiang X, He K, Keegan P, Pazdur R (2016). FDA approval summary: pembrolizumab for the treatment of patients with metastatic non-small cell lung cancer whose tumors express programmed death-ligand 1. Oncologist.

[27] Marcus L, Donoghue M, Aungst S, Myers CE, Helms WS, Shen G (2021). FDA approval summary: entrectinib for the treatment of NTRK gene fusion solid tumors. Clin Cancer Res.

[28] Marcus L, Lemery SJ, Keegan P, Pazdur R (2019). FDA approval summary: pembrolizumab for the treatment of microsatellite instability-high solid tumors. Clin Cancer Res.

[29] Thorsson V, Gibbs DL, Brown SD, Wolf D, Bortone DS, Ou Yang TH (2018). The immune landscape of cancer. Immunity.

[30] Eddy JA, Thorsson V, Lamb AE, Gibbs DL, Heimann C, Yu JX (2020). CRI iAtlas: an interactive portal for immuno-oncology research. F1000Res.

[31] Karunamurthy A, Chauvin J, Morrison R, Bai Y, Sun J, Wang H (2022). Neoadjuvant vidutolimod (vidu) and nivolumab (nivo) results in MPR and immune activation in high-risk resectable melanoma (MEL): final phase II clinical trial results. J Immunother Cancer.

[32] Chesney J, Lewis KD, Kluger H, Hamid O, Whitman E, Thomas S (2022). Efficacy and safety of lifileucel, a one-time autologous tumor-infiltrating lymphocyte (TIL) cell therapy, in patients with advanced melanoma after progression on immune checkpoint inhibitors and targeted therapies: pooled analysis of consecutive cohorts of the C-144-01 study. J ImmunoTher Cancer.

[33] Rottey S, Santoro A, Arnold S, Khan S, Cohn A, Fang B (2022). Cabozantinib plus atezolizumab in advanced head and neck cancer previously treated with platinum-containing chemotherapy: results from cohort 17 of the COSMIC-021 study. J Immunother Cancer.

[34] Foy S, Jacoby K, Bota D, Hunter T, Schoenfeld A, Pa Z (2022). A phase I study of personalized adoptive TCR T cell therapy in patients with solid tumors: safety, efficacy, and T cell trafficking to tumors of non-virally gene edited T cells. J Immunother Cancer.

[35] Ball M, Kremp M, Qureshi R, Sonawane P, Schmierer M, VanDuzer J (2022). Characterization of CT-0508, an anti-HER2 chimeric antigen receptor macrophage (CAR-M), manufactured from patients enrolled in the phase 1, first in human, clinical trial of CT-0508. J Immunother Cancer.

[36] Krebs M, Majem M, Felip E, Forster M, Doger B, Clay T (2020). A phase II study (TACTI-002) of eftilagimod alpha (a soluble LAG-3 protein) with pembrolizumab in PD-L1 unselected patients with metastatic non-small cell lung(NSCLC) or head and neck carcinoma (HNSCC). J Immunother Cancer.

[37] Naing A, Ferrando-Martinez S, Ware M, Haymaker C, Bierly A, Goon J (2022). NT-I7, a long-acting IL-7, plus pembrolizumab favors CD8 T-cell infiltration in liver metastases of heavily pre-treated, immunologically cold, MSS-colorectal and pancreatic cancer. J Immunother Cancer.

[38] Srivastava RM, Trivedi S, Concha-Benavente F, Gibson SP, Reeder C, Ferrone S, Ferris RL (2017). CD137 stimulation enhances cetuximab-induced natural killer: dendritic cell priming of antitumor T-cell immunity in patients with head and neck cancer. Clin Cancer Res.

[39] Ge H, Ferris RL, Wang JH (2023). Cetuximab responses in patients with HNSCC correlate to clonal expansion feature of peripheral and tumor-infiltrating T cells with top T-cell receptor clonotypes. Clin Cancer Res.

[40] Mascarella MA, Olonisakin TF, Rumde P, Vendra V, Nance MA, Kim S (2023). Response to neoadjuvant targeted therapy in operable head and neck cancer confers survival benefit. Clin Cancer Res.

[41] Ferris RL, Spanos WC, Leidner R, Gonçalves A, Martens UM, Kyi C (2021). Neoadjuvant nivolumab for patients with resectable HPV-positive and HPV-negative squamous cell carcinomas of the head and neck in the CheckMate 358 trial. J Immunother Cancer.

[42] Santos P, Kulkarni A, Li H, Chen J, Vujanovic L, Kim S (2022). CD8 T cell repertoire analysis of patients with resectable head and neck cancer enrolled in a phase II neoadjuvant study of α-PD1 administered alone or in combination with α-CTLA4 or α-LAG3. J Immunother Cancer.

[43] Galsky MD, Arija JÁA, Bamias A, Davis ID, De Santis M, Kikuchi E (2020). Atezolizumab with or without chemotherapy in metastatic urothelial cancer (IMvigor130): a multicentre, randomised, placebo-controlled phase 3 trial. Lancet.

[44] Powles T, Csőszi T, Özgüroğlu M, Matsubara N, Géczi L, Cheng SY (2021). Pembrolizumab alone or combined with chemotherapy versus chemotherapy as first-line therapy for advanced urothelial carcinoma (KEYNOTE-361): a randomised, open-label, phase 3 trial. Lancet Oncol.

[45] Powles T, Park SH, Voog E, Caserta C, Valderrama BP, Gurney H (2020). Avelumab maintenance therapy for advanced or metastatic urothelial carcinoma. N Engl J Med.

[46] Powles T, Park SE, Voog E, Caserta C, Valderrama BP, Gurney H (2022). Avelumab first-line (1L) maintenance for advanced urothelial carcinoma (UC): long-term follow-up results from the JAVELIN Bladder 100 trial. J Clin Oncol.

[47] Bajorin DF, Witjes JA, Gschwend JE, Schenker M, Valderrama BP, Tomita Y (2021). Adjuvant nivolumab versus placebo in muscle-invasive urothelial carcinoma. N Engl J Med.

[48] Rosenberg JE, Milowsky M, Ramamurthy C, Mar N, McKay RR, Friedlander T (2022). Study EV-103 Cohort K: antitumor activity of enfortumab vedotin (EV) monotherapy or in combination with pembrolizumab (P) in previously untreated cisplatin-ineligible patients (pts) with locally advanced or metastatic urothelial cancer (la/mUC). Ann Oncol.

[49] Motzer RJ, McDermott DF, Escudier B, Burotto M, Choueiri TK, Hammers HJ (2022). Conditional survival and long-term efficacy with nivolumab plus ipilimumab versus sunitinib in patients with advanced renal cell carcinoma. Cancer.

[50] Choueiri TK, Powles TB, Albiges L, Burotto M, Szczylik C, Zurawski B (2022). Phase III study of cabozantinib (C) in combination with nivolumab (N) and ipilimumab (I) in previously untreated advanced renal cell carcinoma (aRCC) of IMDC intermediate or poor risk (COSMIC-313). Ann Oncol.

[51] Choueiri TK, Powles T, Burotto M, Escudier B, Bourlon MT, Zurawski B (2021). Nivolumab plus cabozantinib versus sunitinib for advanced renal-cell carcinoma. N Engl J Med.

[52] Burotto M, Powles T, Escudier B, Apolo AB, Bourlon MT, Shah AY (2023). Nivolumab plus cabozantinib vs sunitinib for first-line treatment of advanced renal cell carcinoma (aRCC): 3-year follow-up from the phase 3 CheckMate 9ER trial. J Clin Oncol.

[53] Powles T, Plimack ER, Soulières D, Waddell T, Stus V, Gafanov R (2020). Pembrolizumab plus axitinib versus sunitinib monotherapy as first-line treatment of advanced renal cell carcinoma (KEYNOTE-426): extended follow-up from a randomised, open-label, phase 3 trial. Lancet Oncol.

[54] André T, Shiu KK, Kim TW, Jensen BV, Jensen LH, Punt C (2020). Pembrolizumab in microsatellite-instability-high advanced colorectal cancer. N Engl J Med.

[55] Diaz LA, Shiu KK, Kim TW, Jensen BV, Jensen LH, Punt C (2022). Pembrolizumab versus chemotherapy for microsatellite instability-high or mismatch repair-deficient metastatic colorectal cancer (KEYNOTE-177): final analysis of a randomised, open-label, phase 3 study. Lancet Oncol.

[56] Taieb J, Bouche O, André T, Barbier E, Laurent-Puig P, Bez J (2022). Avelumab versus standard second-line treatment chemotherapy in metastatic colorectal cancer (mCRC) patients with microsatellite instability (MSI): The SAMCO-PRODIGE 54 randomised phase II trial. Ann Oncol.

[57] André T, Lonardi S, Wong KYM, Lenz HJ, Gelsomino F, Aglietta M (2022). Nivolumab plus low-dose ipilimumab in previously treated patients with microsatellite instability-high/mismatch repair-deficient metastatic colorectal cancer: 4-year follow-up from CheckMate 142. Ann Oncol.

[58] Chalabi M, Fanchi LF, Dijkstra KK, Van den Berg JG, Aalbers AG, Sikorska K (2020). Neoadjuvant immunotherapy leads to pathological responses in MMR-proficient and MMR-deficient early-stage colon cancers. Nat Med.

[59] Chalabi M, Verschoor YL, van den Berg J, Sikorska K, Beets G, Lent AV (2022). Neoadjuvant immune checkpoint inhibition in locally advanced MMR-deficient colon cancer: The NICHE-2 study. Ann Oncol.

[60] Cercek A, Lumish M, Sinopoli J, Weiss J, Shia J, Lamendola-Essel M (2022). PD-1 blockade in mismatch repair-deficient, locally advanced rectal cancer. N Engl J Med.

[61] Rousseau B, Bieche I, Pasmant E, Hamzaoui N, Leulliot N, Michon L (2022). PD-1 blockade in solid tumors with defects in polymerase epsilon. Cancer Discov.

[62] Bullock AJ, Grossman JE, Fakih MG, Lenz H, Gordon M, Margolin K (2022). Botensilimab, a novel innate/adaptive immune activator, plus balstilimab (anti-PD-1) for metastatic heavily pretreated microsatellite stable colorectal cancer. Ann Oncol.

[63] Fukuoka S, Hara H, Takahashi N, Kojima T, Kawazoe A, Asayama M (2020). Regorafenib plus nivolumab in patients with advanced gastric or colorectal cancer: an open-label, dose-escalation, and dose-expansion phase Ib trial (REGONIVO, EPOC1603). J Clin Oncol.

[64] Morano F, Raimondi A, Pagani F, Lonardi S, Salvatore L, Cremolini C (2022). Temozolomide followed by combination with low-dose ipilimumab and nivolumab in patients with microsatellite-stable, O6-methylguanine-DNA methyltransferase-silenced metastatic colorectal cancer: the MAYA trial. J Clin Oncol.

[65] Mace TA, Shakya R, Pitarresi JR, Swanson B, McQuinn CW, Loftus S (2018). IL-6 and PD-L1 antibody blockade combination therapy reduces tumour progression in murine models of pancreatic cancer. Gut.

[66] Ware MB, Phillips M, McQuinn C, Zaidi MY, Knochelmann HM, Greene E (2023). Dual IL-6 and CTLA-4 blockade regresses pancreatic tumors in a T cell and CXCR3-dependent manner. JCI Insight.

[67] Zhang Y, Ware MB, Zaidi MY, Ruggieri AN, Olson BM, Komar H (2021). Heat shock protein-90 inhibition alters activation of pancreatic stellate cells and enhances the efficacy of PD-1 blockade in pancreatic cancer. Mol Cancer Ther.

[68] Yarchoan M, Cope L, Ruggieri AN, Anders RA, Noonan AM, Goff LW (2021). Multicenter randomized phase II trial of atezolizumab with or without cobimetinib in biliary tract cancers. J Clin Invest.

[69] Schmid P, Adams S, Rugo HS, Schneeweiss A, Barrios CH, Iwata H (2018). Atezolizumab and Nab-paclitaxel in advanced triple-negative breast cancer. N Engl J Med.

[70] Burstein MD, Tsimelzon A, Poage GM, Covington KR, Contreras A, Fuqua SA (2015). Comprehensive genomic analysis identifies novel subtypes and targets of triple-negative breast cancer. Clin Cancer Res.

[71] Szender JB, Papanicolau-Sengos A, Eng KH, Miliotto AJ, Lugade AA, Gnjatic S (2017). NY-ESO-1 expression predicts an aggressive phenotype of ovarian cancer. Gynecol Oncol.

[72] Zamarin D, Burger RA, Sill MW, Powell DJ, Lankes HA, Feldman MD (2020). Randomized phase II trial of nivolumab versus nivolumab and ipilimumab for recurrent or persistent ovarian cancer: an NRG oncology study. J Clin Oncol.

[73] Pujade-Lauraine E, Fujiwara K, Ledermann JA, Oza AM, Kristeleit R, Ray-Coquard IL (2021). Avelumab alone or in combination with chemotherapy versus chemotherapy alone in platinum-resistant or platinum-refractory ovarian cancer (JAVELIN Ovarian 200): an open-label, three-arm, randomised, phase 3 study. Lancet Oncol.

[74] Monk BJ, Colombo N, Oza AM, Fujiwara K, Birrer MJ, Randall L (2021). Chemotherapy with or without avelumab followed by avelumab maintenance versus chemotherapy alone in patients with previously untreated epithelial ovarian cancer (JAVELIN Ovarian 100): an open-label, randomised, phase 3 trial. Lancet Oncol.

[75] Moore KN, Bookman M, Sehouli J, Miller A, Anderson C, Scambia G (2021). Atezolizumab, bevacizumab, and chemotherapy for newly diagnosed stage III or IV ovarian cancer: placebo-controlled randomized phase III trial (IMagyn050/GOG 3015/ENGOT-OV39). J Clin Oncol.

[76] Färkkilä A, Gulhan DC, Casado J, Jacobson CA, Nguyen H, Kochupurakkal B (2020). Immunogenomic profiling determines responses to combined PARP and PD-1 inhibition in ovarian cancer. Nat Commun.

[77] Hamid O, Sato T, Davar D, Callahan MK, Thistlethwaite F, Aljumaily R (2022). Results from phase I dose escalation of IMC-F106C, the first PRAME × CD3 ImmTAC bispecific protein in solid tumors. Ann Oncol.

[78] Dickinson MJ, Carlo-Stella C, Morschhauser F, Bachy E, Corradini P, Iacoboni G (2022). Glofitamab for relapsed or refractory diffuse large B-cell lymphoma. N Engl J Med.

[79] Hammerich L, Marron TU, Upadhyay R, Svensson-Arvelund J, Dhainaut M, Hussein S (2019). Systemic clinical tumor regressions and potentiation of PD1 blockade with in situ vaccination. Nat Med.

[80] Tolcher AW, Brody J, Rajakumaraswamy N, Lakhani NJ, Kuhne MR, Trowe T (2022). Phase 1b study of GS-3583, a novel FLT3 agonist Fc fusion protein, in patients with advanced solid tumors. J Clin Oncol.

[81] Klebanoff CA, Wolchok JD (2018). Shared cancer neoantigens: making private matters public. J Exp Med.

[82] Chandran SS, Ma J, Klatt MG, Dündar F, Bandlamudi C, Razavi P (2022). Immunogenicity and therapeutic targeting of a public neoantigen derived from mutated PIK3CA. Nat Med.

[83] Shoushtari AN, Chatila WK, Arora A, Sanchez-Vega F, Kantheti HS, Rojas Zamalloa JA (2021). Therapeutic implications of detecting MAPK-activating alterations in cutaneous and unknown primary melanomas. Clin Cancer Res.

[84] Chandran SS, Klebanoff CA (2019). T cell receptor-based cancer immunotherapy: emerging efficacy and pathways of resistance. Immunol Rev.

[85] Duerinck J, Schwarze JK, Awada G, Tijtgat J, Vaeyens F, Bertels C (2021). Intracerebral administration of CTLA-4 and PD-1 immune checkpoint blocking monoclonal antibodies in patients with recurrent glioblastoma: a phase I clinical trial. J Immunother Cancer..

[86] Neyns B, Schwarze JK, Bertels C, Geens W, Tijtgat J, Awada G (2021). Intracranial administration of CTLA-4 and PD-1 immune checkpoint blocking monoclonal antibodies in recurrent glioblastoma (rGB): a multi-cohort adaptive phase I clinical trial. Ann Oncol.

[87] Schwarze JK, Geens W, Tijtgat J, Awada G, Seynaeve L, Vanbinst A-M (2022). A phase I clinical trial on intracranial administration of autologous myeloid dendritic cells (myDC) in combination with ipilimumab and nivolumab in patients with recurrent glioblastoma (rGB). J Clin Oncol.

[88] Ahn M, Kim S, Carcereny Costa E, Rodríguez LM, Oliveira J, InsaMolla MA (2022). MEDI5752 or pembrolizumab (P) plus carboplatin/pemetrexed (CP) in treatment-naïve (1L) non-small cell lung cancer (NSCLC): a phase Ib/II trial. Ann Oncol.

[89] Stein MN, Dorff TB, Goodman OB, Thomas RA, Silverman MH, Guo M (2022). A phase 2, multicenter, parallel-group, open-label study of vudalimab (XmAb20717), a PD-1 x CTLA-4 bispecific antibody, alone or in combination with chemotherapy or targeted therapy in patients with molecularly defined subtypes of metastatic castration-resistant prostate cancer. J Clin Oncol.

[90] Missel S, Bunk S, Hofmann M, Pszolla G, Hutt M, Schwoebel F (2022). Targeting solid tumors with IMA402, a next-generation bispecific T cell engaging receptor against PRAME. Ann Oncol.

[91] Bailey SR, Nelson MH, Majchrzak K, Bowers JS, Wyatt MM, Smith AS (2017). Human CD26^high^ T cells elicit tumor immunity against multiple malignancies via enhanced migration and persistence. Nat Commun.

[92] Nelson MH, Knochelmann HM, Bailey SR, Huff LW, Bowers JS, Majchrzak-Kuligowska K (2020). Identification of human CD4+ T cell populations with distinct antitumor activity. Sci Adv.

[93] Galati D, Zanotta S, Capone M, Madonna G, Mallardo D, Romanelli M (2023). Potential clinical implications of CD4^+^CD26^high^ T cells for nivolumab treated melanoma patients. J Transl Med.

[94] Gentles AJ, Newman AM, Liu CL, Bratman SV, Feng W, Kim D (2015). The prognostic landscape of genes and infiltrating immune cells across human cancers. Nat Med.

[95] Khan AN, Kolomeyevskaya N, Singel KL, Grimm MJ, Moysich KB, Daudi S (2015). Targeting myeloid cells in the tumor microenvironment enhances vaccine efficacy in murine epithelial ovarian cancer. Oncotarget.

[96] Singel KL, Emmons TR, Khan ANH, Mayor PC, Shen S, Wong JT (2019). Mature neutrophils suppress T cell immunity in ovarian cancer microenvironment. JCI Insight.

[97] Emmons TR, Giridharan T, Singel KL, Khan ANH, Ricciuti J, Howard K (2021). Mechanisms driving neutrophil-induced T-cell immunoparalysis in ovarian cancer. Cancer Immunol Res.

[98] Markiewski MM, DeAngelis RA, Benencia F, Ricklin-Lichtsteiner SK, Koutoulaki A, Gerard C (2008). Modulation of the antitumor immune response by complement. Nat Immunol.

[99] Ajona D, Ortiz-Espinosa S, Moreno H, Lozano T, Pajares MJ, Agorreta J (2017). A combined PD-1/C5a blockade synergistically protects against lung cancer growth and metastasis. Cancer Discov.

[100] Faje AT, Lawrence D, Flaherty K, Freedman C, Fadden R, Rubin K (2018). High-dose glucocorticoids for the treatment of ipilimumab-induced hypophysitis is associated with reduced survival in patients with melanoma. Cancer.

[101] Arbour KC, Mezquita L, Long N, Rizvi H, Auclin E, Ni A (2018). Impact of baseline steroids on efficacy of programmed cell death-1 and programmed death-ligand 1 blockade in patients with non-small-cell lung cancer. J Clin Oncol.

[102] Bai X, Hu J, Betof Warner A, Quach HT, Cann CG, Zhang MZ (2021). Early use of high-dose glucocorticoid for the management of irAE is associated with poorer survival in patients with advanced melanoma treated with anti-PD-1 monotherapy. Clin Cancer Res.

[103] Hughes MS, Molina GE, Chen ST, Zheng H, Deshpande V, Fadden R (2019). Budesonide treatment for microscopic colitis from immune checkpoint inhibitors. J Immunother Cancer.

[104] Lee MD, Seethapathy H, Strohbehn IA, Zhao SH, Boland GM, Fadden R (2021). Rapid corticosteroid taper versus standard of care for immune checkpoint inhibitor induced nephritis: a single-center retrospective cohort study. J Immunother Cancer.

[105] Luoma AM, Suo S, Wang Y, Gunasti L, Porter CBM, Nabilsi N (2022). Tissue-resident memory and circulating T cells are early responders to pre-surgical cancer immunotherapy. Cell.

[106] Blum SM, Zlotoff DA, Smith N, Ramesh S, Kernin L, Sen P (2022). Single-cell profiling of human heart and blood in immune checkpoint inhibitor-associated myocarditis. J Clin Oncol.

[107] Patrinely JR, Johnson R, Lawless AR, Bhave P, Sawyers A, Dimitrova M (2021). Chronic immune-related adverse events following adjuvant anti-PD-1 therapy for high-risk resected melanoma. JAMA Oncol.

[108] Drobni ZD, Alvi RM, Taron J, Zafar A, Murphy SP, Rambarat PK (2020). Association between immune checkpoint inhibitors with cardiovascular events and atherosclerotic plaque. Circulation.

[109] Park BC, Lee AXT, Ye F, Turker I, Johnson DB (2022). Immune checkpoint inhibitors and their impact on liver enzymes and attenuation. BMC Cancer.

[110] Wu B, Chiang HC, Sun X, Yuan B, Mitra P, Hu Y (2020). Genetic ablation of adipocyte PD-L1 reduces tumor growth but accentuates obesity-associated inflammation. J Immunother Cancer.

[111] Patrinely JR, Young AC, Quach H, Williams GR, Ye F, Fan R (2020). Survivorship in immune therapy: assessing toxicities, body composition and health-related quality of life among long-term survivors treated with antibodies to programmed death-1 receptor and its ligand. Eur J Cancer.

[112] Lee NY, Ferris RL, Psyrri A, Haddad RI, Tahara M, Bourhis J (2021). Avelumab plus standard-of-care chemoradiotherapy versus chemoradiotherapy alone in patients with locally advanced squamous cell carcinoma of the head and neck: a randomised, double-blind, placebo-controlled, multicentre, phase 3 trial. Lancet Oncol.

[113] Saddawi-Konefka R, O'Farrell A, Faraji F, Clubb L, Allevato MM, Jensen SM (2022). Lymphatic-preserving treatment sequencing with immune checkpoint inhibition unleashes cDC1-dependent antitumor immunity in HNSCC. Nat Commun.

[114] Altorki NK, McGraw TE, Borczuk AC, Saxena A, Port JL, Stiles BM (2021). Neoadjuvant durvalumab with or without stereotactic body radiotherapy in patients with early-stage non-small-cell lung cancer: a single-centre, randomised phase 2 trial. Lancet Oncol.

[115] Ban Y, Markowitz GJ, Zou Y, Ramchandani D, Kraynak J, Sheng J (2021). Radiation-activated secretory proteins of Scgb1a1+ club cells increase the efficacy of immune checkpoint blockade in lung cancer. Nat Cancer.

[116] Wei J, Montalvo-Ortiz W, Yu L, Krasco A, Ebstein S, Cortez C (2021). Sequence of αPD-1 relative to local tumor irradiation determines the induction of abscopal antitumor immune responses. Sci Immunol..

[117] Wilkins A, Fontana E, Nyamundanda G, Ragulan C, Patil Y, Mansfield D (2021). Differential and longitudinal immune gene patterns associated with reprogrammed microenvironment and viral mimicry in response to neoadjuvant radiotherapy in rectal cancer. J Immunother Cancer.

[118] Bifulco C, Piening B, Bower T, Robicsek A, Weerasinghe R, Lee S, et al. Identifying high-risk breast cancer using digital pathology images. Nightingale Open Sci. 2021. 10.48815/N5159B.

[119] Kehl KL, Xu W, Gusev A, Bakouny Z, Choueiri TK, Riaz IB (2021). Artificial intelligence-aided clinical annotation of a large multi-cancer genomic dataset. Nat Commun.

[120] Rakaee M, Adib E, Ricciuti B, Sholl LM, Shi W, Alessi JV (2023). Association of machine learning-based assessment of tumor-infiltrating lymphocytes on standard histologic images with outcomes of immunotherapy in patients With NSCLC. JAMA Oncol.

